# The cholera toxin B subunit induces trained immunity in dendritic cells and promotes CD8 T cell antitumor immunity

**DOI:** 10.3389/fimmu.2024.1362289

**Published:** 2024-05-15

**Authors:** Araceli Tepale-Segura, Julián A. Gajón, Samira Muñoz-Cruz, Octavio Castro-Escamilla, Laura C. Bonifaz

**Affiliations:** ^1^ Unidad de Investigación Médica en Inmunoquímica, Unidad Médica de Alta Especialidad (UMAE) Hospital de Especialidades, Centro Médico Nacional Siglo XXI, Instituto Mexicano del Seguro Social, Mexico City, Mexico; ^2^ Instituto Politécnico Nacional, Escuela Nacional de Ciencias Biológicas, Departamento de Inmunología, Mexico City, Mexico; ^3^ Posgrado en Ciencias Bioquímicas, Facultad de Química, Universidad Nacional Autónoma de México, Mexico City, Mexico; ^4^ División de Investigación Clínica, Coordinación de Investigación en Salud, Centro Médico Nacional Siglo XXI, Instituto Mexicano del Seguro Social, Mexico City, Mexico; ^5^ Coordinación de Investigación en Salud, Centro Médico Nacional Siglo XXI, Instituto Mexicano del Seguro Social, Mexico City, Mexico

**Keywords:** trained immunity, dendritic cells, CTB adjuvant, CD8 T cells, melanoma

## Abstract

**Introduction:**

Innate immune training is a metabolic, functional, and epigenetic long-term reprogramming of innate cells triggered by different stimuli. This imprinting also reaches hematopoietic precursors in the bone marrow to sustain a memory-like phenotype. Dendritic cells (DCs) can exhibit memory-like responses, enhanced upon subsequent exposure to a pathogen; however, whether this imprinting is lineage and stimulus-restricted is still being determined. Nevertheless, the functional consequences of DCs training on the adaptive and protective immune response against non-infectious diseases remain unresolved.

**Methods:**

We evaluated the effect of the nontoxic cholera B subunit (CTB), LPS and LTA in the induction of trained immunity in murine DCs revealed by TNFa and LDH expression, through confocal microscopy. Additionally, we obtained bone marrow DCs (BMDCs) from mice treated with CTB, LPS, and LTA and evaluated training features in DCs and their antigen-presenting cell capability using multiparametric cytometry. Finally, we design an experimental melanoma mouse model to demonstrate protection induced by CTB-trained DCs in vivo.

**Results:**

CTB-trained DCs exhibit increased expression of TNFa, and metabolic reprogramming indicated by LDH expression. Moreover, CTB training has an imprint on DC precursors, increasing the number and antigen-presenting function in BMDCs. We found that training by CTB stimulates the recruitment of DC precursors and DCs infiltration at the skin and lymph nodes. Interestingly, training-induced by CTB promotes a highly co-stimulatory phenotype in tumor-infiltrating DCs (CD86+) and a heightened functionality of exhausted CD8 T cells (Ki67+, GZMB+), which were associated with a protective response against melanoma challenge in vivo.

**Conclusion:**

Our work indicates that CTB can induce innate immune training on DCs, which turns into an efficient adaptive immune response in the melanoma model and might be a potential immunotherapeutic approach for tumor growth control.

## Introduction

Paradigmatically, innate immunity is responsible for first-line defense against pathogens and priming the adaptive response to elicit adequate clearance of these agents. Interestingly, the innate cells can also display immune memory features upon a secondary stimulation due to intrinsic changes, thereby maintaining a long-term functional memory that challenges the traditional immune memory model (1–3). Unlike adaptive immune memory, which relies on gene rearrangement and clonal expansion, innate immune memory, known as trained immunity, is based on epigenetic and metabolic rewiring mechanisms that enhance the responsiveness of innate immune cells to a wide range of secondary non-specific stimuli (1, 4–6). The epigenetic reprogramming in trained innate immune cells is sustained by histone post-transcriptional modifications at the promoters of genes encoding pro-inflammatory cytokines such as TNFα (7–10). This epigenetic landscape also affects chromatin accessibility to regulate the transcription of molecules associated with innate function (11–13). Thus, the heightened expression of these molecules is considered a hallmark of trained immunity (12, 14).

Different metabolic pathways are essential to sustain the pro-inflammatory phenotype in trained cells, and increased glycolysis is the primary metabolic pathway associated with this phenotype (15). Indeed, high glucose intake and further transformation to ATP via anaerobic glycolysis are crucial to maintaining striking transcriptional activity in the trained cells (15, 16). Consequently, higher pyruvate conversion to lactic acid by lactate dehydrogenase (LDH) is widely observed in innate immune cells and represents another hallmark of trained immunity (16, 17). In addition, trained cells exhibit enhanced responses against identical or heterologous secondary stimulus, lasting at least three months, regardless of the short lifespan of innate immune cells, suggesting hematopoietic stem cell (HSC) involvement (18–20). Indeed, BCG immunization in mice and humans promotes the reprogramming of HSC precursors towards innate cell lineages (18, 21). In line with this, lipopolysaccharide (LPS) stimulation through TLR4/CEBPβ signaling induces training features on long-term HSC, that are transcriptionally cryptic until a secondary inflammatory stimulation (22). Nevertheless, this training is not achieved with LTA (lipoteichoic acid) stimulation, delineating that not all TLR ligands are training inducers. Other evidence shows that bone marrow transference from BCG or β-glucan-trained mice into naïve mice results in increased inflammatory response in myeloid mature cells after homologous or heterologous stimulation (19, 21, 23, 24).

The first evidence of innate immune training was observed in the monocyte/macrophage lineage (9, 23, 24); however, it has also been reported that dendritic cells (DCs) can acquire training features (25). DCs are a cell lineage that encloses a heterogeneous group of professional antigen-presenting cells (APCs) with specific functional abilities to active naïve T cells. Classical type 1 DCs (cDCs1) are masters of antigen cross-presentation to CD8 T cells (26–28), while Classical type 2 DCs (cDCs2) are specialists in priming CD4 T cells (28, 29). In mice, a particular subset of DCs, named inflammatory dendritic cells (InfDCs), are derived from Ly6C^high^ monocytes under particular inflammatory stimuli, such as *L. monocytogenes* infection, and are prone to prime either CD4 or CD8 T cells (30–32).

The induction of trained immunity in DCs might help optimize immunotherapies strategies for better clearance of infections or even in cancer treatment. Indeed, growing evidence suggests that trained immunity is beneficial for long-term protection against recurrent pathogen exposure and is an auxiliary protection mechanism in vaccination schemes (1, 14). Although many vaccines use antigens of the target pathogen plus an adjuvant to achieve an effective response, the possibility of using adjuvants capable of promoting trained immunity has not been evaluated. We have previously described that the non-toxic beta subunit of cholera toxin (CTB), a safe and well-tolerated adjuvant, robustly activates skin dendritic cells for at least seven days and this extended activation induces long-lasting memory in CD4 Th1 and Th17 cells (33). In addition, we recently showed that CTB is an adjuvant in prophylactic immunization in a B16 melanoma model, promoting substantial infiltration of tissue-resident memory CD8 T cells (34). Notably, in the tumor microenvironment, the majority of CD8 infiltrating cells exhibit an exhausted phenotype as a result of chronic antigen stimulation and lack of positive co-stimulatory signals. In this context, the phenotype of DCs has been recognized as a potential regulator of lymphocyte T exhaustion (35). However, it is unclear whether the CTB’s long-lasting and robust activation on DCs is associated with the induction of trained phenotype in these cells and the repercussion over the generation of protective adaptive immune response.

In this context, this work aimed to determine whether CTB could train murine DCs and their impact on the adaptive response. We evaluated the expression of TNFα to assess the DCs-enhanced inflammatory function and upregulation of LDH for metabolic rewiring, as well as the expression of CD86, a critical molecule for proper co-stimulation of T cells (36, 37). We next investigated if the CTB stimulation has an imprint on DC precursors, finding an increase in pre-DC and BMDC numbers and function. We determined the functional consequence of DCs training in a tumor context, where a narrow communication between DCs and CTLs is necessary for optimal antitumoral immunity (38, 39). Pre-stimulation of DCs with CTB induced higher expression of TNFα and LDH after second stimulation *in vitro* and *in vivo*, arguing for training phenotype. Furthermore, these trained DCs exhibited higher CD86 expression, and the CTB-trained mice displayed an effective antitumor immunity against melanoma challenge, associated with a robust infiltration of highly co-stimulatory DCs and functional CD8 T cells. These findings reveal that CTB is a training stimulus able to induce an efficient protective immune response in a tumor context.

## Materials and methods

### Mice

Eight-week-old C57BL/6 mice were obtained from the Experimental Medicine Unit facility of the National Autonomous University of Mexico (UNAM) or purchased from Bioinvert Company® (CDMX, Mexico). The experiments were conducted following the Institutional Ethics Committees and the Mexican national regulations. This work was approved by a scientific committee of the Instituto Mexicano del Seguro Social with registration number R-2020-785-004.

### Mouse inoculation with CTB, LPS or LTA

All stimuli were inoculated intradermally in both ears of each mouse by injecting half of the total inoculum on each ear. One group of mice was inoculated with 10 µg of CTB (Sigma Aldrich), and after 3, 7, or 14 days post-inoculation, the mice were euthanized, and the ear skin was collected to analyze the cell suspensions. A second group of mice was inoculated with 10 µg of CTB or PBS (vehicle), and after 14 days, they were re-stimulated with the same dose of CTB or vehicle. After 7 days post-stimulation, the ears and the skin-draining lymph nodes (dLN) were collected to obtain cell suspensions. In the Bone Marrow derived DCs experiments, mice were inoculated with 10 µg of CTB, 10 µg of LPS (Invivogen), 200 µg of purified LTA (Sigma Aldrich), or vehicle (PBS). After 14 days (resting time) post-inoculation, mice were euthanized, and the femurs and tibias were collected to obtain bone marrow cell suspensions. For *in situ* experiments, mice were inoculated with 10 µg of CTB, 10 µg of LPS (Invivogen), or 200 µg of purified LTA (Sigma Aldrich). After 14 days, mice were re-stimulated with 10 µg of CTB, and 3 days later, ears were collected to obtain tissues, which were fixed in 4% paraformaldehyde and embedded in paraffin for histological analysis.

### Cell-suspension preparation from skin and skin-draining lymph nodes

The lymph nodes and the ears were treated with 0.25 mg/mL liberase™ TL (Thermolysin low) (Roche) and 0.125 mg/mL DNAse (Roche) at 37°C for 25 minutes and 45 minutes, respectively. Tissues were chopped and incubated at the same conditions for 45 min more under constant shaking. Next, enzymatic digestion was stopped by adding 0.5μM EDTA, and cell suspensions were filtered through a 70 μm strainer (Corning). Afterwards, cells were washed with RPMI-1640 (Biowest) digestion medium containing 10% Fetal Bovine Serum (FBS) (Biowest), 2 mM L-glutamine (Corning), 100 IU Penicillin, and 100 μg/mL Streptomycin (Corning), by centrifugation at 400 g for 5 minutes. Then, 0.125 mg/mL DNAse was added, and cells were washed with digestion media for 5 minutes at 400 g. The supernatant was discarded, and cells were counted or stained, as needed.

### Generation of mouse bone marrow-derived dendritic cells (BMDCs)

Bone marrow was obtained from the femurs and tibias of 8-week-old C57/BL6 mice after inoculation with different stimuli, as previously mentioned. The femurs and tibias were obtained free of muscle tissue and placed in PBS pH 7.4, the epiphyses were cut and the bone marrow from both bones was extracted by successive irrigations with supplemented RPMI-1640 medium (10% FBS (Biowest), 2mM L-glutamine (Corning), and penicillin-streptomycin solution (Corning), using a 1 mL syringe with a 25G 5/8 needle. The cell suspensions were filtered through nylon mesh and then washed twice with supplemented RPMI-1640 medium and counted using a Neubauer chamber. To generate DCs, 5 x 10^6^ bone marrow cells were cultured in 8 mL of supplemented RPMI-1640 medium containing 10% of cell culture supernatant from recombinant GM-CSF-producing CHO (hamster ovary cells). The cell cultures were maintained at 37°C and 5% CO_2_, adding fresh medium with GM-CSF every third day. The BMDCs were harvested on day 7 and used for different experiments.

### BMDCs maturation and OT-I T cell cocultures

After seven days of differentiation, 5 x 10^6^ BMDCs were stimulated with 1 μg/mL of LPS and harvested 24 hours later to analyze their activation status and phenotype whereas control cells were incubated with the culture medium. Additionally, differentiated BMDCs were pulsed with OVA (100 μg/mL) for 30 minutes and then stimulated with 1 μg/mL LPS for 24 hours. Afterward, BMDCs were co-cultured with Cell Trace Violet (CTV)-labeled OT-I cells at a 1:10 of DCs to CD8 cells ratio, for 5 days. After this time, both cells were harvested and analyzed as needed.

### Immune cell characterization by flow cytometry

Cell surface staining for DCs was performed by incubating cell suspensions for 20 minutes at 4°C with the following mixture of antibodies: anti-MHC-II (I-A/IE)-FITC (clone: NIMR4), anti-CD11c-PE-CF5 (clone: HL3C), anti-CD86-PE (clone: PO3.1), (all from eBioscience); anti-CD103-PECy7 (clone: 2E7), anti-CD11b-BV510 (clone: M1/70), anti-Ly6C-APC/Cy7 (clone: HK1.4), lineage negative-PERCP anti: [(CD3 (clone: 17A2), CD19 (clone: 6D5), TER119 (clone: TER-119), CD49b (clone: DX5)]; anti-Flt3-PE (clone: A2F10), anti-SIRPα-PE (clone: SE5A5), and anti-CD45-APC (clone: T3/2.3) (all from Biolegend); Live/Dead Fixable Violet (Thermo Fisher). After cell surface staining, cells were fixated and permeabilized using the fixation and permeabilization buffer set (Thermo Fisher), according to the manufacturer’s instructions. After that, the cell suspension was incubated with anti-TNFα-PE (clone: 2E7, Biolegend) for 30 minutes at room temperature and then washed three times with PBS for 5 minutes at 400 g. To perform cell count, a fraction of cells was stained with anti-CD45-PECy7 (clone:30-F11, Biolegend) and DAPI (Thermo Fisher) and then immediately mixed with CountBright™ absolute counting beads (Thermo Fisher). Finally, all cell suspensions were resuspended in fluorescence-activated cell sorting (FACS) buffer and analyzed on a BD FACS Canto II or BD FACS ARIA IIu or BD Influx™ (Becton, Dickinson and company). Flow cytometry data were analyzed using the FlowJo software V10.8 (Tree Star, Inc.).

For BMDCs characterization, cell surface staining for DCs was performed by incubating cell suspensions for 20 minutes at 4°C with the following mixture of antibodies: Live-or-Dye 568-583 (Biotium), anti-CD11c-PE-CF594 (clone: HL3C), anti-MHC-II (I-A+I-E)-FITC (clone: NIMR4), anti-CD11b-BV510 (clone: M1/70), anti-CD103-PECy7 (clone: 2E7), anti-CD45-BV750 (clone:30-F11), anti-Ly6C-APC/Cy7 (clone: HK1.4), anti-CD86-BV480 (clone: GL-1), anti-CD40-Super Bright 780 (clone:1C10), anti-PD-L1-BV711 (clone:10F.9G2), anti-CCR7-APC-Fire 810 (clone:4B12), anti-Flt3-PE (clone: A2F10), anti-SIRPα-APC (clone: O12), anti-MHC-I (H-2kb+H-2Db)-BV42, lineage negative-PERCP anti: [(CD3 (clone: 17A2), CD19 (clone: 6D5), TER119 (clone: TER-119), CD49b (clone: DX5)]; anti-Flt3-APC (clone: A2F10), anti-SIRPα-PE (clone: SE5A5), and anti-CD45-APC (clone: T3/2.3) (all from Biolegend). After cell surface staining, cells were fixated and permeabilized using the fixation and permeabilization buffer set (Thermo Fisher), according to the manufacturer’s instructions. After that, the cell suspensions were incubated with anti-TNFα-PE (clone: 2E7, Biolegend) for 60 minutes at room temperature and then washed twice with PBS for 5 minutes at 400 g. Cell suspensions were resuspended in PBS and acquired on a spectral cytometer Cytek Aurora (Cytek®, Biosciences).

For CD8^+^ T cells characterization after co-culture with BMDCs, cell suspensions were incubated for 20 minutes with Live-or-Dye 568-583 (Biotium), anti-CD3-PERCP (clone:17A2), anti-CD8α-APC7/Cy7 (clone: 53-6), anti-CD103-PE-Cy7 (clone:2E7), anti-PD-1-PE-Fire 640 (clone:29F.1A12), anti-TIM3-PE-Dazzle 594 (clone: B8.2C12), anti-CD69-PE (clone: H1.2F3), anti-CD45-BV750 (clone: 30-F11), anti-CD28-BV711 (clone:37.51) (All from Biolegend). After cell surface staining, cells were fixated and permeabilized using the fixation and permeabilization buffer set (Thermo Fisher), according to the manufacturer’s instructions. The cell suspension was incubated with anti-TNFα-Alexa Fluor 660 (clone: MP6-XT22), anti-TCF1-Alexa Fluor 488 (clone: C63D9), anti-Ki67-BV650 (clone:11F6), anti-IFNγ-PE-Alexa Fluor 610 (clone: XMG1.2) (All from Biolegend) and anti-Granzyme B (GZMB)-Alexa Fluor 700 (Clone: QA16A02, B.D Biosciences) for 60 minutes at room temperature. Then, cells were washed twice with PBS for 5 minutes at 400 g, resuspended in PBS, and data were acquired on a spectral cytometer Cytek Aurora (Cytek®, Biosciences).

To identify the tumor-infiltrating CD8 T cells, cell suspensions were incubated for 20 minutes at 4°C with the following mixture of antibodies: anti-CD8α-APC7/Cy7 (clone: 53-6.7, Biolegend), anti-SLAMF6-Brilliant Violet 711 (clone:13G3, B.D. Biosciences) anti-CD69-PE (clone: H1.2F3), anti-CD44-BV510 (clone: IM7), anti-CD103 -PE/Cy7 (clone: 2E7-4), anti-CD45-BV750 (clone: 30-F11) (All from Biolegend) and Live/Dead Fixable Violet (Thermo Fisher). After cell surface staining, cells were fixated and permeabilized using the True Nuclear Factor set (Biolegend), according to the manufacturer’s instructions. After that, the cell suspensions were incubated with anti-TCF1-Alexa Fluor 488 (clone: Rabbit mAb, Cell Signaling Technology), anti-GZMB-Alexa Fluor 700 (Clone: QA16A02, B.D Biosciences), anti-PD-1-APC (clone: 29F.1A12, Biolegend), anti-Ki67-BV605 (clone: 16A8 Biolegend), anti-Tim3-PE/Dazzle 594 (clone: B8.2C12), Biolegend) for 45 minutes at room temperature and then washed three times with PBS for 5 minutes at 400g. Cell suspensions were resuspended in PBS, and data were acquired on a BD Influx™ (Becton, Dickinson and company).

### Flow cytometry high dimensional data analysis

Data sets from CD11c^+^ MHC-II^+^ and CD8^+^ events (approximately 10,000 for each subset), with adjusted compensation parameters, were exported to individual files, and then, concatenated to perform a dimensionality reduction with the t-distributed stochastic neighbor embedding (t-SNE) algorithm, with the corresponding plugin from the CD11c, MHC-II, CD103, CD11b, Ly6c, and CD86 markers for DCs. Data visualization was based on the Barnes Hut gradient algorithm with exact KNN, using the following parameters: perplexity value of 30, iterations values of 1,000, and a learning rate of 180,000. t-SNE graphs were generated using the Flowjo 10.8 software (Tree Star, Ashland, OR, United States).

For Uniform Manifold Approximation and Projection (UMAP) unsupervised clustering, singlets, live, CD45^+^, lineage^-^, CD11^High^ MHC-II^High^, were concatenated from all samples with all compensated parameters at 2500 events per sample and concatenated (45 000 total events) as Flow Cytometry Standard (FCS) files. UMAP clusterization was performed with all compensated parameters (CCR7, MHC-I, PD-L1, CD40, CD86, TNFα, CD103, CD11b, Ly6c, FLT3 and SIRPα) except for, viability, CD45^+^, lineage^-^, CD11^High^, MHC-II^High^. We used Euclidean approximation with 15 nearest neighbors, a minimum distance of 0.5, and 2 components. An unsupervised clustering map was performed with the FlowSOM plugin algorithm using the same compensated parameters to perform an unsupervised clustering map.

### Analysis by confocal microscopy

To determine training-induced metabolic changes *in situ*, 3μm thick sections from paraffin-embedded tissues obtained during the aforesaid procedures were placed on charged glass slides (Superfrost Plus Yellow), and re-hydrated in serial solutions of 100% Xylene, 50/50 Xylene/EtOH, 80% EtOH, 50% EtOH and Mili-Q water. Antigen retrieval was performed in citrate buffer pH 6.0 (sodium citrate 10 μM) at 90°C for 20 min. Tissue sections were permeabilized for 2 hours (bovine serum albumin 10 mg/mL, horse serum 5%, Triton 0.3%, and sodium azide 0.02%) and incubated for 18 hours with the following primary antibodies: anti-CD11c (clone: N418, Biolegend); anti-CD86 (clone: PO3.1, eBioscience), anti-TNFα (clone: 2E7, Biolegend), and anti-LDH (polyclonal ab47010, Abcam). Tissues were washed five times with PBS and then incubated for 2 hours with the following secondary mAb, Anti-hamster IgG (H+L)-Alexa Fluor 488 (Jackson ImmunoResearch Lab), anti-rat IgG (H+L)-Alexa Fluor 594 (Jackson ImmunoResearch Labs), and anti-rabbit IgG (H+L)-Alexa Fluor 647 (Jackson ImmunoResearch Labs). The stained tissues were examined with an Eclipse Ti inverted confocal microscope (Nikon Corporation) using NIS Elements v.4.50. Imaging was performed using a 20x (dry, NA 0.8) objective lens. Zoom was performed at 3.4x. Images were analyzed using FIJI ImageJ Software (ImageJ software, National Institutes of Health).

### Melanoma cell line culture

The B16-F10-OVA melanoma cell line (MO4) (SCC420 Merck Millipore) was cultured in DMEM medium supplemented with 10% FBS, 0.1% penicillin-streptomycin, 0.2% L-glutamine, 0.05% 2-mercaptoethanol, 0.01% sodium pyruvate, 0.1% HEPES and 0.1% non-essential amino acids (all from Biowest), at 37°C and 5% CO_2_ until they reached 95% of confluence. Then, cells were harvested and used for tumor induction in mice.

### Melanoma mouse model

Mice were inoculated intradermally with 10 μg of CTB (Sigma Aldrich) and 14 days later re-stimulated with the same amount of CTB plus 1μg anti-DEC/OVA. After seven days, melanoma tumors were established by subcutaneous injection of 5.0 x 10^5^ MO4 cells in the left flank. The width and length of tumors were measured every third day from day 3 until day 21. Tumor appearance was scored every third day via manual palpation, and the tumor volume (mm^3^) was calculated as 4/3π (1/2 width)^2^ (1/2 length) in mm^3^. The final tumor mass was assessed from the tumors excised on the last day of the challenge. Mice with no evidence of tumor at the end of the period were scored as tumor-free.

### Tumor cell suspensions

The tumors were harvested at day 21 and analyzed by flow cytometry. Tumor-infiltrating lymphocytes (TILs) were obtained using a previously described method (26). Briefly, the tumor was finely minced and incubated with 400(U/mL) Collagenase D (Roche) and (5 μg/mL) DNAse (Roche) for 1 hour at 37°C. Next, enzymatic digestion was stopped by adding 0.5μM EDTA, and digested tissues were filtered through a 70μm strainer (Corning). After that, the cell suspensions were treated with 0.2 mg/mL DNase. The lymphocyte interface of the centrifuged Percoll 40/90 solution was recovered, washed, and stained as needed.

### Statistics

The statistical significance was calculated using one-way ANOVA with Tukey’s comparison to quantify populations, and Kruskal Wallis with Dunn´s Comparison to Mean Fluorescence Intensity (MFI) in more than two groups and tumor-free percentage was calculated using the Log-rank (Mantel-Cox) test. A p-value of *< 0.05, **< 0.01, ***< 0.001, ****< 0.0001 was considered as significant difference. All analyses were performed using Prism 6.0 (GraphPad Software Inc., La Jolla, CA, USA).

## Results

### CTB promotes the infiltration and activation imprint of different DC subsets in the skin

**Figure 1:**
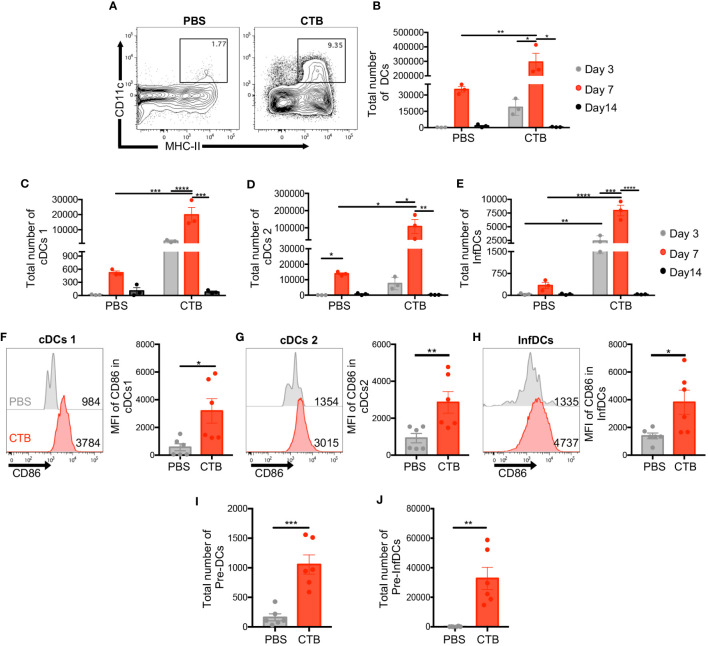
Intradermal CTB administration increases the number of DC precursors and three DC subsets with activated phenotype, in the skin. C57BL6 mice were inoculated intradermally (i.d.) with 10 μg of CTB in the ears (5 μg for each ear) or PBS (vehicle). Cells isolated from the ear skin at 3, 7, and 14 days post-stimulation were stained for characterization and quantification by flow cytometry. **(A)** Representative contour plots of total DCs (CD11c^+^ MHC-II^+^) after 7 days of stimulation. Kinetics of the total number of **(B)** DCs (CD11c^+^ MHC-II^+^), and three DC subsets **(C)** cDCs1 (CD103^+^ CD11b^-^), **(D)** cDCs2 (Ly6c^-^ CD11b^+^), and **(E)** InfDCs (Inflammatory DCs) (Ly6c^+^ CD11b^+^). The results are shown as Mean ±SEM (n=3). **(F–H)** Representative histograms and bar graphs of the Mean Fluorescence Intensity (MFI) of CD86 expression in **(F)** cDCs1, **(G)** cDCs2, and **(H)** InfDCs in the skin 7 days after each treatment. Bar graphs of the total number of **(I)** conventional Pre-DCs and **(J)** inflammatory DC precursors (Pre-InfDCs), phenotyped in the skin after 7 days of treatment. The results of CD86 expression and the total number of pre-DCs and pre-InfDCs are shown as Mean ±SEM (n=6) and pooled from 2 independent experiments. Statistical Analysis: one-way ANOVA with Tukey’s comparison test and Kruskal Wallis with Dunn´s Comparison test. * p<0.05 **p< 0.005, ***p = 0.0001 ****p<0.0001.

Previously, our group showed DCs activation by CTB (33). To determine the impact of this adjuvant on DC phenotype and subsets, skin cell suspensions were obtained from CTB-inoculated mice and analyzed by flow cytometry at 3, 7, and 14 days post-inoculation. The skin DCs were characterized as viable, CD45^+^, lineage^-^, CD11c^+^ MHC-II^+^ (**Supplementary Figure 1A**). We confirmed that CTB promoted an increase in the total DCs (CD11c^+^, MHC-II^+^) proportion (**Figure 1A**) and number, which reached a peak on day 7 and diminished on day 14 (**Figure 1B**). In addition, we determined the activation state by evaluating the expression of CD86 on DCs surface. **Supplementary Figure 1B** shows that the expression of CD86 in DCs from mice treated with CTB is significantly higher compared to control mice treated with PBS. Considering the changes observed in total skin DCs, we evaluated whether dermal subsets of cDCs1, cDCs2, and inflammatory DCs (InfDCs) could be affected by CTB administration, using the CD11b, CD103, and Ly-6C markers (**Supplementary Figures 1A, C**). CTB induced an increased proportion of cDCs1 (CD103^+^, CD11b^-^), cDCs2 (CD11b^+^, Ly6c^-^), and InfDCs (CD11b^+^, Ly-6C^+^) compared to mice treated with PBS (**Supplementary Figure 1C**). Interestingly, after 3, and 7 days post CTB inoculation, we observed the recruitment of cDCs1, cDCs2, and InfDCs, while on day 14 a significant decrease was observed in all subsets, as well as in total DCs (**Figures 1B–E**). Regarding the activation state, it was observed that CTB administration also induced a high expression of CD86 in all DC subsets compared to the control group (**Figures 1F–H**). It has been proposed that under inflammatory conditions, DC precursors from peripheral blood can migrate to the skin and give rise to DC subsets (40). To evaluate if CTB increases DC precursors, we used the markers FLT3, SIRPα, and Ly-6C (**Supplementary Figure 2A**) and determine their frequency. In a steady state, there are few classical DC precursors (pre-DCs) and inflammatory dendritic cell precursors (pre-InfDCs) in the skin. In contrast, after CTB stimulation the percentage and the absolute number of both precursors were significantly increased (**Figures 1I, J**, **Supplementary Figures 2B, C**). These results indicate that CTB promotes the recruitment of DCs and their precursors and induces an activated phenotype on skin DCs.

### CTB *in vivo* recall unveils the induction of trained immunity *in situ* by metabolic, functional, and phenotypic changes on skin DCs

**Figure 2:**
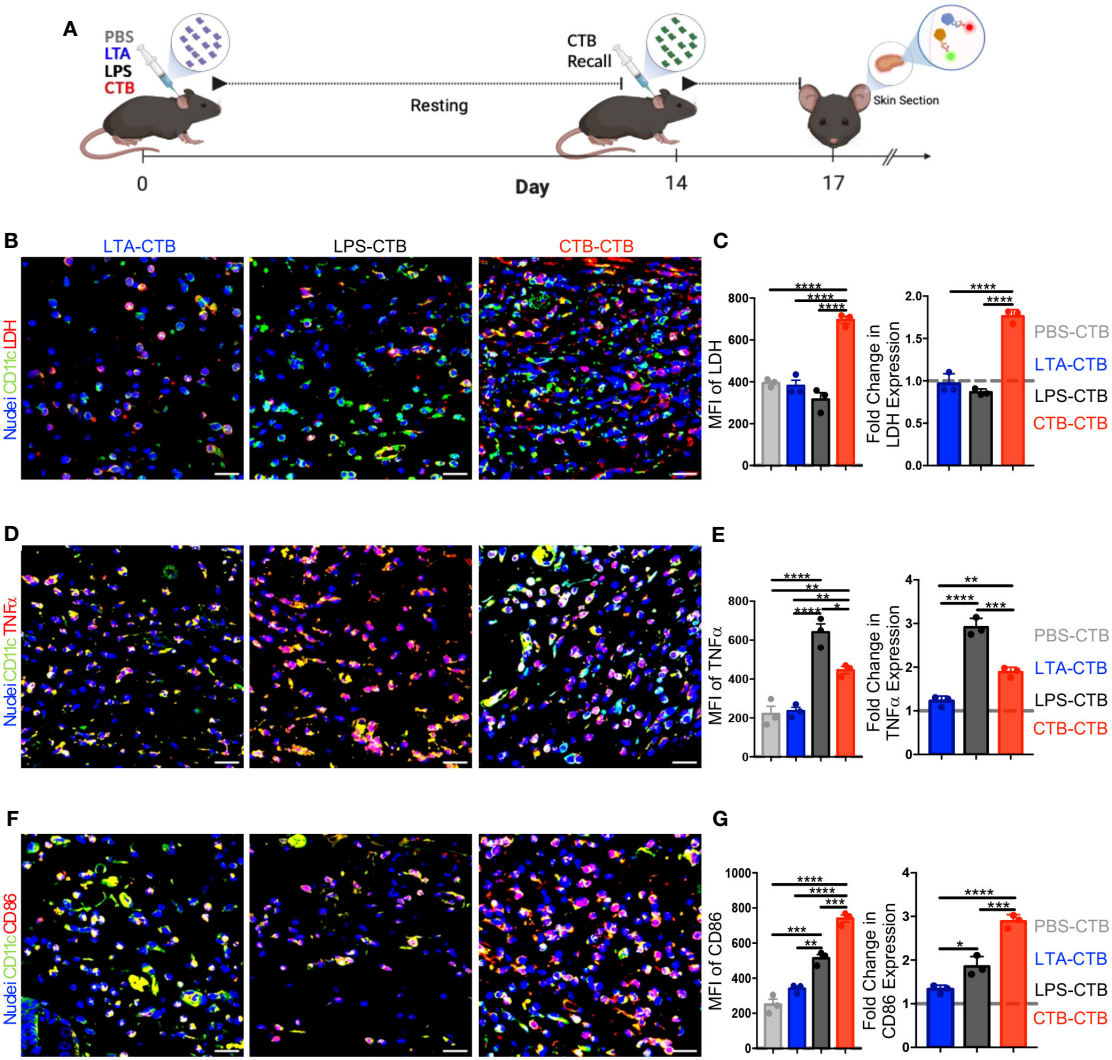
CTB restimulation *in vivo* induces metabolic, functional, and phenotypic changes associated with trained immunity on skin DCs. **(A)** Schematic representation of the experimental design. C57BL6 mice were inoculated intradermally (i.d.) with 10 μg of CTB, or 10 µg of LPS, or 200 µg of purified LTA, or PBS (vehicle). After a resting time of 14 days, the animals were boosted with 10 μg of CTB and sacrificed 3 days after to obtain skin sections. Representative microscopy micrographs of **(B)** DCs CD11c^+^ (green) and LDH (red), **(D)** DCs CD11c^+^ (green) and TNFα (red), **(F)** DCs CD11c^+^ (green) and CD86 (red) for each treatment (Scale bar 20 μm). The bar graphs show the mean fluorescence intensity (MFI) for each group (left panel) and the Fold-change in the MFI compared to the PBS-CTB treatment (right panel), for the expression of **(C)** LDH, **(E)** TNFα, and **(G)** CD86. The dotted grey line indicates the expression of LDH, TNFα and CD86 with a single CTB administration as a reference. Spots represent the Mean of MFI obtained from three areas of the tissues in 3 mice per group. The results are shown as Mean±SEM (n=3). Statistical analysis: one-way ANOVA with Tukey’s comparison test **P < 0.005, and ****P<0.0001. Mouse model figure created with Biorender. *P<0.05 and ***P=0.0001.

Trained immunity is characterized by the heightened production of pro-inflammatory cytokines by innate immune cells in response to a second stimulus equal to or different from the initial one (2, 11, 14). To determine whether an adjuvant such as CTB, which sustains DCs activation, could induce trained immunity features in DCs such as the expression of LDH, TNFα, and CD86, we designed a mouse model to compare CTB stimulation with two other inflammatory stimuli LPS, which induces trained immunity, and LTA, which does not (22). The impact of these stimuli on skin DC training induction was evaluated *in situ* at day 17 post-primary inoculation (3 days after the CTB re-stimulation and the 14 days of resting) (**Figure 2A**).

First, we evaluated the kinetic of DCs activation, after stimulation with a single dose of each stimulus, and compared them to PBS (unstimulated control) (**Supplementary Figures 3A, B**). The results showed a different DCs kinetic activation between the three stimuli. For instance, LTA and LPS induced a DCs strong expression of CD86, TNFα, and LDH at 24 hours (**Supplementary Figures 3B–D**); however, this expression returned to basal levels at day 3 post-stimulation (**Supplementary Figures 3C, D, F, G**). The functional and metabolic activation of DCs induced by CTB, determined by the expression of CD86 and TNFα, was similar to LPS and LTA after 24 hours. Nevertheless, this activation was maintained for 7 days and returned to basal levels at day 14 (**Supplementary Figures 3E, H**). Collectively, these results strongly suggest that DCs stimulated with LTA, LPS, or CTB are in a functional and metabolic steady state at day 14 (resting time) (**Supplementary Figures 3F–H**).

To evaluate the induction of training by CTB compared to LTA or LPS, we determined the expression *in situ* of LDH, CD86, and TNFα after 3 days of the CTB recall for each stimulus, through confocal microscopy. The findings revealed that LDH expression after the CTB recall in LTA and LPS treatments was not different from a single CTB stimulation; in contrast, the double CTB stimulation induced significantly higher LDH expression (**Figures 2B, C**). Accordingly, a positive synergistic effect (≈2-fold increase) was observed after CTB recall in the LDH expression, in the mice with a previous CTB administration compared to those with a single CTB administration (**Figure 2C**). Moreover, the TNFα expression, a classic cytokine associated with the immune trained phenotype, or CD86, a co-stimulatory molecule associated with DCs activation, was not increased after LTA treatment and the CTB recall. However, we observed an important increase in TNFα and a slight increase in the CD86 expression in the mice that received LPS as the first stimulus and with CTB recall (**Figures 2D, E**). Remarkably, there was a significant increase in the intensity and fold change expression of TNFα and CD86 after the CTB recall, in mice that received CTB as the first stimulus (**Figures 2F, G**). Taken together, these results strongly suggest that stimulation with CTB induces trained immunity in skin DCs, which is associated with the increased expression of LDH, TNFα, and CD86 upon secondary exposure to CTB and after a period of metabolic rest.

### CTB training has an imprint on DC precursors increasing pre-DCs and BMDCs number and function

**Figure 3:**
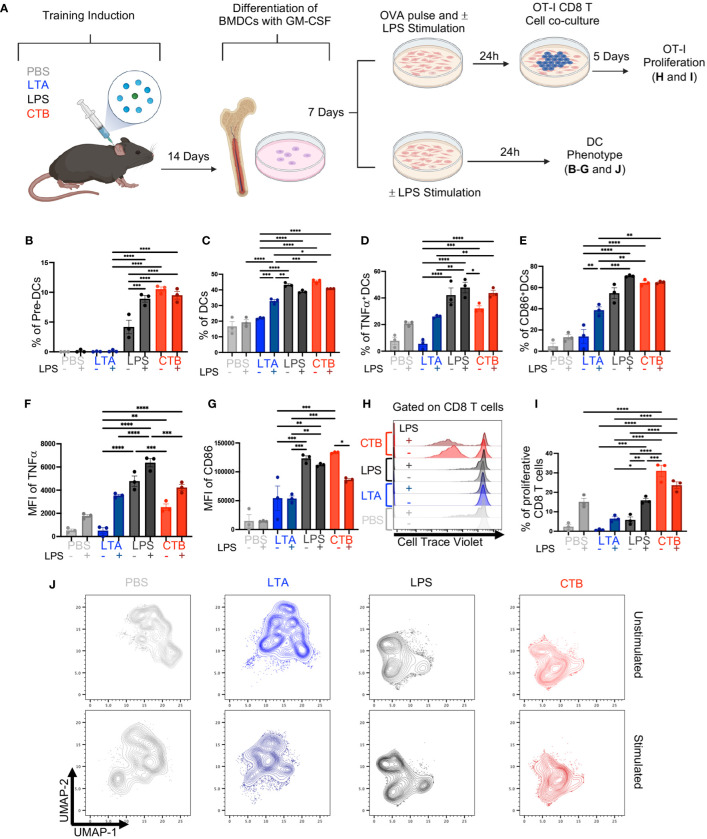
CTB administration promotes trained immunity in DCs and DC precursors, enhancing the antigen-presenting function of DCs. **(A)** Schematic representation of the experimental design. C57BL6 mice were inoculated intradermally (i.d.) with 10 μg of CTB or 10 µg of LPS or 200 µg of purified LTA or PBS (vehicle). After 14 days, bone marrow cells were obtained and differentiated to DCs (BMDCs) with GM-CSF for 7 days. BMDCs were pulsed with OVA (100 μg/mL) and stimulated (+) or not (-) with 1 μg/mL of LPS, for 24 hours and then co-cultured with OT-I T cells in a 1:10 ratio, for 5 days. Afterward, the cells were harvested to evaluate the T cell phenotype and proliferation by spectral cytometry. Additionally, BMDCs were stimulated (+) or not (-) with 1 μg/mL of LPS for 24 hours, harvested and characterized by spectral cytometry. Bar graphs of the percentage of **(B)** Pre-DCs, **(C)** DCs, **(D)** TNFα^+^ DCs and **(E)** CD86^+^ DCs (Mean ± SEM, n=3), for each treatment, and the Mean Fluorescence Intensity (MFI) for **(F)** TNFα and **(G)** CD86 expression for each condition. **(H)** Representative histograms for T cell proliferation for each stimulus. **(I)** Bar graph of the percentage of proliferative CD8^+^ T cells co-cultured with BMDCs previously pulsed with OVA and stimulated (+) or not (-) with LPS. **(J)** Uniform Manifold Approximation and Projection (UMAP) plots showing dimensionality reduction and clustering, among concatenated groups; without LPS (unstimulated) (upper panels) and with LPS (stimulated) (bottom panels) for each condition. Data from pooled bone marrow cells of three mice per stimulus, in triplicate for each condition. Statistical analysis: one-way ANOVA with Tukey’s comparison test **p < 0.005, ***p<0.001, and ****p<0.0001. Mouse model figure created with Biorender. *P<0.05.

Innate immune training relies on hematopoietic stem cell reprogramming of myeloid precursors, which sustain a trained phenotype in differentiated cells, such as monocytes (18, 19). Hence, to determine whether CTB immune training observed in skin DCs, could also occur in hematopoietic precursors, we derived DCs from the bone marrow (BM) of mice inoculated with either PBS, LTA, LPS, or CTB, after 14 days of resting and evaluated their activation and capability to induce CD8 T cell response. The obtained BM cells were cultured for 7 days with GM-CSF, and the half of BMDCs were stimulated or not with LPS for 24 hours, whereas the other half were pulsed with OVA in the presence or absence of LPS, and then co-cultivated with OT-I CD8 T cells for 5 days (**Figure 3A**) and evaluated by flow cytometry as shown in **Supplementary Figure 4A**.

We found a high percentage of pre-DCs in the LPS-treated condition, which increased after LPS stimulation. We also observed a high frequency of pre-DCs in mice treated with CTB in absence or presence of LPS (**Figure 3B**), in contrast to those treated with PBS or LTA in which pre-DCs were not observed. Furthermore, we found a higher proportion of DCs differentiated from CTB or LPS-treated mice compared to PBS or LTA conditions, in the presence or absence of LPS (**Figure 3C**). Interestingly, we observed a high percentage of BMDCs TNFα^+^ subset under LPS and CTB conditions, even without secondary stimulation (**Figure 3D**). We only observed increased frequencies of TNFα<σπ>+</σπ> DCs and CD86^+^ DCs when DCs derived from PBS and LTA were stimulated with LPS (**Figures 3D, E**). LPS treatment induced a higher expression of TNFα compared to CTB treatment, with a slight increase after LPS re-stimulation (**Figure 3F**, **Supplementary Figure 4C**). Additionally, we found that both CTB and LPS conditions induced similar expression of CD86 on BMDCs, which was higher than LTA or PBS treatment, independent of LPS stimulation (**Figure 3G**, **Supplementary Figure 4C**). Regarding the T cell activation, we observed a slight OT-I proliferation in PBS, LTA, or LPS conditions after OVA pulse with LPS stimulation. Remarkably, we observed a robust T cell proliferation and IFNγ production in CTB-trained BMDCs pulsed with OVA, even in the absence of LPS, compared to the other experimental conditions (**Figures 3H, I**, **Supplementary Figures 4B, D, E**), suggesting that CTB training could be associated with antigen cross-presentation in DCs.

Finally, we performed a non-supervised analysis with additional markers on DCs such as CCR7, CD40, PDL1, and MHC-I to discern deeper phenotypic changes. We observed that trained conditions (LPS and CTB) were segregated into different clusters in the UMAP plot, indicating significant phenotypic changes compared to non-trained conditions (**Figure 3J** upper panels). In addition, trained BMDCs re-stimulated with LPS *in vitro* did not segregate into new clusters in the UMAP. However, this stimulation reinforced these clusters (**Figure 3J** bottom panels). Interestingly, training-associated clusters displayed a higher expression of TNFα and CD86 (**Supplementary Figure 4F**). Altogether, these results demonstrate that CTB can induce immune training at bone marrow precursors level, and increase antigen presentation function on DCs, as revealed by a higher induction of functional proliferating CD8 T cells, suggesting a differential impact on T cell response compared to other training inductors, such as LPS.

### Trained Immunity by CTB promotes robust protection against melanoma *in vivo*

**Figure 4:**
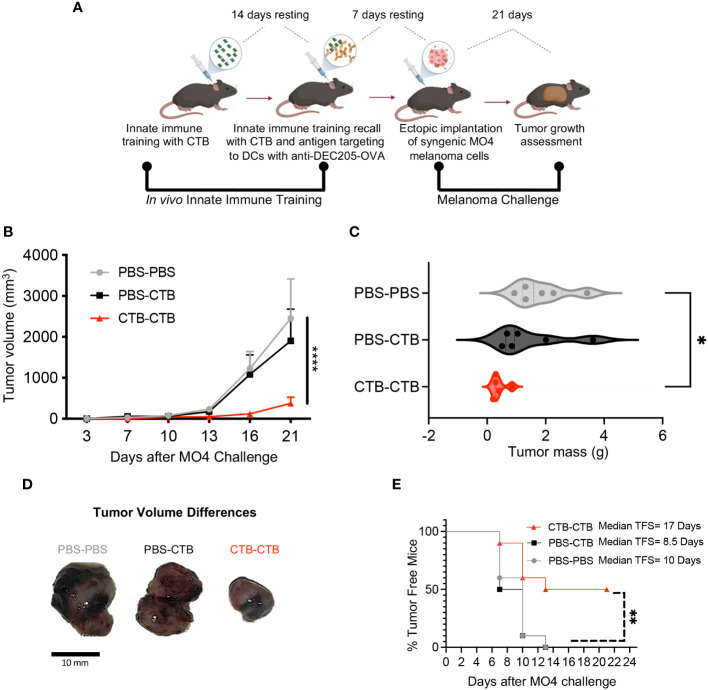
Innate immune training induced by CTB on murine DCs favors antitumor response against melanoma challenge. **(A)** Experimental design to induce *in vivo* innate immune training and the subsequent tumor challenge. C57BL/6 mice were administered via i.d. with 10 µg of CTB in the ears (5 μg for each ear) or PBS (vehicle), and 14 days later (resting time), were inoculated with the same dose of CTB or PBS, plus anti-DEC205-OVA conjugated antibody (1 μg). After 7 days of resting time, mice were subcutaneously (s.c.) challenged with MO4 melanoma cells, and tumor growth was assessed every 3 days until day 21, when mice were sacrificed, and tumors collected and weighed. **(B)** Tumor growth curve and **(C)** Violin plot comparing tumor mass among the groups (Mean ± SEM is shown), **(D)** Representative images of tumors at the endpoint (day 21). **(E)** Percentage of tumor-free mice in all groups and estimated median tumor-free survival (TFS) for each group. Data were pooled from two independent experiments (n=5-6 per group). Statistical analysis: **(B, C)** One-way ANOVA with Tukey´s multiple comparison test *p<0.05, ****p< 0.0001, **(D)** Log-Rank (Mantel-Cox) test Chi square= 11.32, p=0.003. Mouse model figure created with Biorender. **P<0.01.

The impact of DCs immune training on adaptive immunity has been scarcely explored. Our results showed an increase in CD86 expression in skin CTB-trained DCs and BMDCs, along with higher induction of proliferation in OT-I CD8 T cells, indicating an improvement in the antigen-presenting function of these cells after CTB training. To determine if these effects were associated with CTB inflammation or training induction, we established a training CTB recall model to distinguish the inflammatory response from the trained response in DCs at a local and systemic level. For this aim, we obtained DCs from the skin (**Supplementary Figure 1A**) and the skin-draining lymph nodes (dLN) (**Supplementary Figure 5A**) after seven days of CTB recall. We observed that a resting period of 14 days after the training stimulus, followed by 7 days after training recall, was suitable to differentiate CTB inflammation from the CTB-trained response. This was demonstrated by the presence of infiltrating DCs and pre-DCs in the skin (**Supplementary Figures 5B–D**) and migratory DCs with an activated phenotype into dLN (**Supplementary Figures 5G–I**). Besides, we observed that trained cells in the skin reached draining lymph nodes and showed an enhanced inflammatory response and co-stimulatory capacity, similar to that observed at the inoculation site (**Supplementary Figures 5E, F, J, K**). Next, we explored the effect of inflammation (one CTB inoculation) versus training (double CTB inoculation) in a murine melanoma model where strong cooperation of DCs with CD8 T cells is required to control tumor growth. We trained mice with a first stimulation with CTB *in vivo*, and 14 days later recalled this innate training with a second CTB stimulation together with an anti-DEC205/OVA targeted antibody to deliver ovalbumin antigen to DCs. After 7 days of CTB recall, we challenged the trained mice with B16-OVA (MO4) melanoma cells subcutaneously (s.c), and we assessed tumor growth dynamics (**Figure 4A**). We observed significantly higher protection against melanoma tumor growth in the innate immune-trained mice (CTB-CTB) compared with the mice treated only with PBS (PBS-PBS), or mice that were pre-treated with PBS and received CTB and anti-DEC205/OVA (PBS-CTB) as secondary stimuli (**Figure 4B**).

Moreover, we observed a significantly lower tumor mass in the CTB-trained mice compared with the untrained mice, suggesting an important role of innate immune training in tumor growth control (**Figure 4C**). These differences are clearly illustrated in **Figure 4D**, we observed that the tumors arising from trained mice were smaller than in control groups. Finally, we calculated tumor-free survival among the groups and observed a significant protection against tumor development in trained mice compared to control groups. We found that CTB-induced training extended tumor-free survival almost two-fold compared with the other experimental conditions (17 days in CTB-CTB, 8.5 days in PBS-CTB, and 10 days in PBS-PBS) (**Figure 4E**). These results unveiled a notable protection against melanoma challenge *in vivo*, derived from innate immune training induced in dendritic cells by CTB.

### Protection against melanoma is associated with tumor-infiltrating trained classical and inflammatory dendritic cells with a highly co-stimulatory phenotype

**Figure 5:**
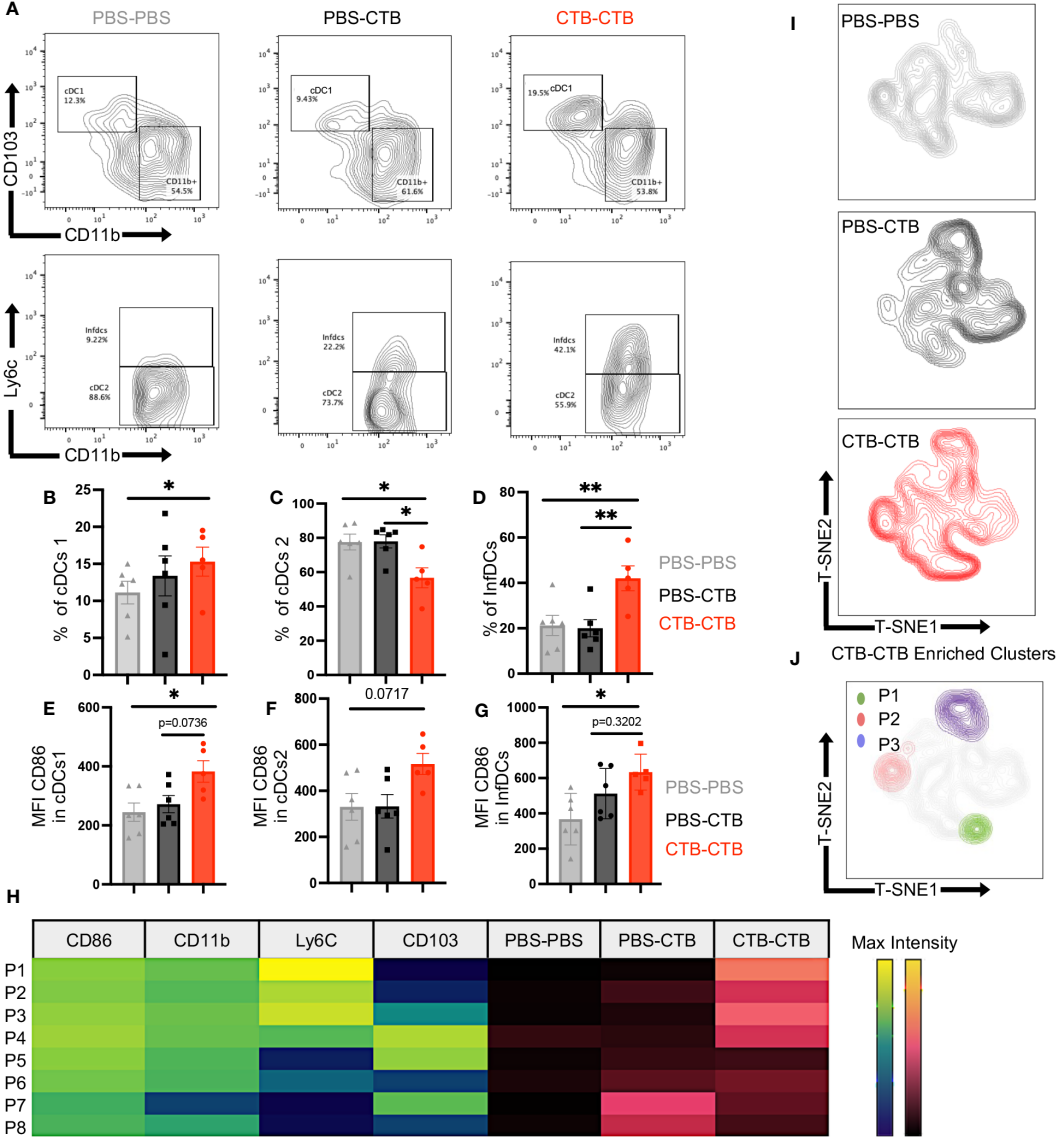
Innate immune training induces infiltration of classical and inflammatory DCs with a highly costimulatory phenotype in melanoma tumors. Tumor-infiltrating cells were harvested from tumors obtained as previously described in the mouse model in **Figure 4A**. Subsets and phenotypes of infiltrating DCs were evaluated by multiparametric flow cytometry. Representative contour plots of **(A)** cDCs1 (CD103^+^ CD11b^-^) (upper panels) and cDCs2 (CD11b^+^ Ly6c^-^) or InfDCs (CD11b^+^ Ly6C^+^) (Bottom panels). Bar graphs showing the percentage of **(B)** cDC1, **(C)** cDCs2, and **(D)** InfDCs, and Mean Fluorescence Intensity (MFI) of CD86 in **(E)** cDCs1, **(F)** cDCs2, and **(G)** InfDCs. **(H)** Heatmap showing the MFI (Viridis Scale) of CD86, CD11b, Ly6c and CD103 on FlowSom differentially enriched clusters from t-SNE dimensionality reduction (P1-P8) and its abundance among treatments (Magma Scale). **(I)** t-SNE plots show dimensionality reduction and clustering among concatenated groups, and **(J)** FlowSom clusters (P1-P3) differentially enriched in CTB-CTB training conditions. Data were pooled from two independent experiments (n=5-6 mice per group). Mean ±SEM One-way ANOVA with Tukey´s multiple comparison test *p<0.05, **p<0.01.

Next, we asked if the robust protection against melanoma challenge observed in immune-trained mice could be associated with changes in dendritic cell phenotype and infiltration in MO4 melanoma tumors. Hence, we obtained tumor-infiltrating DCs (**Supplementary Figures 6A, B**, **Figure 4A**) and analyzed the percentage of total DCs and their three subsets cDCs1, cDCs2, and InfDCs, as well as CD86 expression among subsets. We did not observe changes in the percentage of total DCs between the groups (**Supplementary Figure 7A**). Nonetheless, we found a significant increase in the percentage of cDC1s in CTB-trained mice compared with control groups (**Figures 5A** upper panels, **5B**). Moreover, there was a significant decrease of cDC2 (**Figures 5A** bottom panels, **5C**) that was accompanied by an increase of InfDCs in innate immune-trained mice compared with control groups (**Figures 5A** bottom panels, **5D**).

Furthermore, all the subsets analyzed presented a highly co-stimulatory phenotype in CTB-trained mice, according to higher expression of CD86, and no changes in MHC-II expression in DC subsets compared with control groups (**Figures 5E–G**, **Supplementary Figures 7B, C**).

Finally, we perform a dimensionality reduction among total DCs in all groups using t-SNE algorithm to get unbiased phenotypical differences in DCs (**Supplementary Figure 7D**). Unsupervised clusterization and FlowSom algorithm showed differentially enriched clusters (P1-P8) from t-SNE dimensionality reduction (**Figure 5H**). Furthermore, we observed that CTB-CTB enriched clusters were associated with trained DCs, characterized by a high expression of CD86 (**Figure 5H**, **Supplementary Figure 7E**). In addition, at least three clusters, hereafter referred to as P1, P2, and P3, carrying a highly co-stimulatory phenotype and high expression of either Ly6C, CD11b, or CD103, were exclusively found in the trained condition (**Figures 5I, J**). These results indicate that CTB training achieves long-lasting imprinting in the DCs lineage promoting the control of tumor growth.

### Innate immune training on dendritic cells augments the functionality of exhausted CD8^+^ T cells infiltrating melanoma tumors

**Figure 6:**
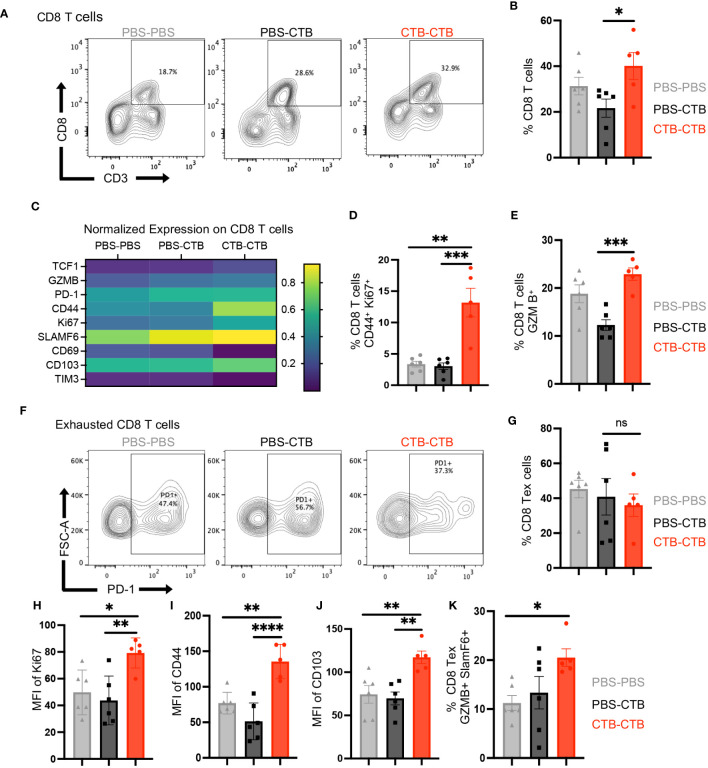
Innate immune training on DCs induces a robust antitumor response through a function enhancement of CD8^+^ tumor-infiltrating T cells. Tumor infiltrating cells were harvested from tumors obtained according to **Figure 4A**, and the phenotype and function of infiltrating CD8^+^ T cells were evaluated by multiparametric flow cytometry. **(A)** Representative dot plots of CD3^+^ CD8^+^ T cells, and **(B)** Percentage of CD8^+^ from total CD45^+^ cells, for each condition. **(C)** Heatmap of function and phenotype markers expression on CD8^+^ T cells, among treatments. Bar graphs of the percentage of **(D)** CD44^+^ Ki67^+^ CD8 T cells and **(E)** Granzyme B^+^ (GZMB) CD8^+^ T cells. **(F)** Representative dot plots of Exhausted CD8^+^ T (Tex) cells PD-1^+^ among groups. **(G)** Bar graphs showing the percentage of Tex (PD-1^+^ CD8^+^) T cells among groups. **(H)** The percentage of Tex Ki67^+^ cells. The Bar graphs show the Mean Fluorescence Intensity (MFI) of **(I)** CD44 and **(J)** CD103, in exhausted CD8^+^ T cells. **(K)** Percentage of exhausted CD8^+^T cells SLAMF6^+^ Granzyme B^+^. Data were pooled from two independent experiments (n=5-6 mice per group). Mean ±SEM One-way ANOVA with Tukey´s multiple comparison test *p<0.05, **p<0.01, ***p<0.001. ****P<0.0001.

One of the major drivers of immune escape from tumor cells is the progressive loss of functionality by CD8**^+^** T cells, named exhaustion. This functional state has been associated with chronic antigen stimulation and scarce co-stimulatory signals. It has been described that narrow contact between antigen-presenting cells and exhausted CD8**^+^** T cells in tumor stroma is associated with good prognosis and therapeutic response. Considering the phenotype variations in co-stimulatory molecule expression on trained DC subsets, we next evaluated the phenotype changes on infiltrating CD8**^+^** T cells by multiparametric flow cytometry, particularly in exhausted CD8**^+^** T cells (**Supplementary Figure 8**). we found a significant increase in the percentage of CD8^+^ T cells in CTB-trained mice compared to control groups (**Figures 6A, B**). Moreover, extensive phenotyping in CD8^+^ T cells from tumors showed higher expression in TCF1, Granzyme B (GZMB), CD44, Ki67, PD-1, and CD103 (**Figure 6C**). Furthermore, innate immune-trained mice exhibited superior infiltration of a highly proliferative memory CD8^+^ T cells (CD44^+^, Ki67^+^) compared to control groups (**Figure 6D**), together with a greater proportion of cytotoxic CD8**^+^** T cells (**Figure 6E**). Next, we assessed if this functional enhancement was influenced by a reinvigoration of exhausted CD8**^+^** T cells (CD8**^+^** Tex). There were no significant changes in the proportion of CD8**^+^** Tex cells among the different groups (**Figures 6F, G**). However, tumor-infiltrating CD8**^+^** Tex cells in trained mice displayed a higher expression of Ki67, CD44, and CD103 (**Figures 6H–J**), indicating that trained condition was associated with a functional and proliferative reinvigoration on CD8^+^ Tex cells. Finally, tumors from trained mice exhibited a higher proportion of SLAMF6^+^ and Granzyme B^+^ CD8 ^+^T ex cells (**Figure 6K**) that resembled a functional exhausted precursor phenotype. Overall, these results demonstrate an association between infiltrating trained DCs and the reinvigoration of CD8**^+^** T cells in tumors.

## Discussion

Innate immune training has redefined the understanding of how innate immune cells respond against subsequent stimuli, such as chronic inflammatory diseases, vaccination, or cancer (38, 39, 41, 42). The functional repercussion of immune training on innate cells, such as DCs, and its potential use as an immunotherapeutic approach are a current point of discussion (6, 14, 42, 43). In this work, we demonstrate that CTB, a cholera toxin-derived adjuvant, can induce trained immunity features in DCs, revealed by the heightened expression of TNFα and LDH, after CTB-recall *in vivo*. Furthermore, this training has an imprint on BM precursors increasing pre-DCs and DCs number and function. In addition, CTB training promotes the recruitment of pre-DCs at the stimulation site and the migration of activated DCs into dLN. Notably, CTB-trained immunity has an impact on the T cell response, which protects mice against melanoma challenge. This effect was associated with a robust infiltration of cDC1 and InfDCs, which highly expressed CD86 in melanoma tumors. Concomitantly with this trained phenotype, tumor-infiltrating CD8 T cells displayed a highly functional and Tex-reinvigorated phenotype, arguing for a beneficial impact of DCs training in a tumor context.

Trained immunity can be triggered by distinct stimuli, including microbial agents, which can protect against heterologous lethal infections (2, 6, 12, 44). In humans, BGC vaccination has demonstrated robust protection against unrelated bacterial and viral infections such as yellow fever, pneumonia, and SARS-COV-2 (44–46). Here, we have evaluated the use of CTB as a potential inductor of trained immunity in DCs because it has proven to be an adjuvant that promotes efficient adaptive immunity response (33, 34). Interestingly, we demonstrated that intradermal stimulation with CTB promoted the activation of skin DCs by increasing the expression of TNFα and CD86; which is consistent with previous findings of our group (33, 47). We also observed that CTB inoculation activates conventional DCs and InfDCs, showing that this adjuvant has a vast range capability to activate several DCs subsets. InfDCs had previously been observed only in response to specific inflammatory stimuli such as the infection of *L. monocytogenes* (48–50). Notably, we showed that intradermal administration of CTB can induce the recruitment of InfDCs, indeed, only a few adjuvants that can induce a robust activation of DCs in a non-invasive administration route are available for clinical use (51), including CTB (52, 53).

Several studies have demonstrated that trained monocytes and macrophages increase the production of inflammatory cytokines, such as TNFα, in response to a secondary inflammatory stimulus (54, 55); which has been scarcely explored in dendritic cells (25). Furthermore, this enhanced response observed in innate trained cells was evaluated after the resting period to avoid confusion with chronically activated phenotype in innate cells (56). Our results showed that dendritic cells trained with CTB required a resting period of 14 days, in which TNFα, LDH, and CD86 returned to their basal expression, which was different compared with other inflammatory stimuli such as LPS or LTA, that required 3 days to return at the basal state, these data suggest that CTB has different activation properties in innate cells. Interestingly, our findings demonstrated that immune training produced by CTB enhanced the expression of TNFα *in situ* similar to LPS. However, CTB training promoted higher LDH expression than LPS, pointing to differential metabolic rewiring among training stimuli. CTB training also showed higher induction of CD86 compared to LPS, which reinforces the idea of a differential training induction regarding metabolic adaptation and antigen presentation function in CTB-trained DCs. Related to this, we observed a strong OT-I CD8 T cell proliferation after co-culture with CTB-trained BMDCs pulsed with OVA, indicating that training induced by CTB increases antigen presentation function on DCs. This could be associated with a higher cross-presentation capacity on CTB-trained DCs since OT-I cells recognize OVA peptides in the context of MHC-I molecules, particularly SIINFEKL peptide (57). However, deeper changes in APC biology shaped by the CTB-induced innate immune training must be explored. Remarkably, immune training on innate hematopoietic precursors has been identified as a potential mechanism for long-term protection mediated by trained cells, however, how the training stimuli could impact the bone marrow is not fully understood (18, 19, 21, 58).

Regarding this, de Laval et al. have delineated that LPS but not LTA could imprint long-term term-HSC by direct stimulation of TLR4/CEBPβ pathway, proposing a mechanism of innate immune training in HSCs, by TLR4 ligands (22). Interestingly, we observed that CTB, which can also be recognized by TLR4 (59), induced training in bulk DC precursors, showed by an enhanced expression of TNFα in BMDCs from CTB-trained mice, which was similar to LPS-trained mice. Additionally, this increased expression that was even observed without LPS stimulation could be explained by the resting time (14 days) or by an activation signaling induced by GM-CSF during BMDCs differentiation, as previously reported (60, 61). On the other hand, cryptic changes derived from innate immune training have been observed in early differentiated bone marrow cells, such as LT-HSC. Therefore, the non-cryptic changes observed in the present report may suggest differential transcriptional regulation derived from innate immune training, which depends on the differentiation state of DC precursors.

The co-stimulatory signals from DCs, such as CD86, are essential for T cell activation and function (62–64), our results highlighted the augmented capacity to stimulate T cells after CTB training. Indeed, this co-stimulatory signal has taken relevance in different chronic inflammatory pathologies, bacterial infections, and cancer because the continuous activity of the CD28/CD86 axis may prevent T cell dysfunction and promote correct antigen clearance at the inflammatory site (65–67). Thus, it is possible that CTB-trained DCs could help treat these conditions considering the capability to induce higher expression of CD86. Moreover, we observed that CTB immune training in the skin was reflected in dLN, where we observed a robust infiltration of migratory dendritic cells expressing TNFα and CD86, arguing for a local and systemic repercussion of training since in dLN DCs activate and differentiate naïve T cells (68–70).

Related to this, we demonstrated that innate immune training with CTB followed by administration of antigen OVA targeted to DCs by anti-DEC205, protected mice against B16-OVA tumor growth and extended tumor-free survival time up to7 days, suggesting a protective role of DCs training in murine melanoma. This might be explained by metabolic adaptation driven by CTB training (increased LDH expression), which could prepare DCs for a highly hypoxic milieu in a tumor context. Indeed, lactate metabolism is usually associated with mitochondrial dysfunction in innate cells and has been proposed as a metabolic barrier within the tumor context (71, 72), this mechanism could be overcome by CTB training. On the other hand, the control of tumor growth could be a result of a proper antitumoral response from T cells elicited by CTB-trained DCs with high expression of CD86. Regardless of that trained stimuli and tumor inoculation were spatiotemporally separated, strengthening the notion that innate immune training could exert systemic protection.

As a significant functional repercussion of training, we demonstrated that CTB training induced a higher infiltration of DCs CD86^+^ in the tumor, particularly of cDCs1, the best APCs to CD8 T cells (73), suggesting that immune training could promote the protection in melanoma model by rewiring cDCs infiltration and activated phenotype. Moreover, this systemic protection could be related to CTB training in DC precursors since we observed imprinting in BMDCs with higher T cell priming capacity after CTB training. These results suggest that CTB could induce trained immunity via TLR-4 on DC precursors but also by a TLR- independent mechanism on cDC1 considering the lack of TLR-4 expression in this subset (36, 74, 75).

Related to this, we observed a robust mobilization of pre-DCs at the inoculation site and into dLN in CTB-trained mice, suggesting a distribution of trained BM precursors to peripheral tissues, where pre-DCs differentiate into trained DCs which could improve the immune response in the tumor. In addition, our results from trained mice show that InfDCs could protect mice against melanoma because these cells robustly infiltrated trained mice highly expressing CD86. In this respect, studies have described that InfDCs participate in the induction of antitumor immunity by promoting the differentiation of CD8^+^ T lymphocytes. in a positive feedback axis IFNγ-IL-12 (32, 76). Nonetheless, this axis could be sustained by cDC1 (77, 78) and could reflect a type 1 polarized phenotype in CTB-trained DCs that positively impacts the tumor growth control (73), potentially by a higher induction of IFNγ in tumor-infiltrating lymphocytes, as we observed in OT-I co-cultures with CTB trained BMDCs. Training on DCs impacted the functional shape of CD8 T cells infiltrating melanoma tumors, which displayed the expression of Ki67 and Granzyme B, associated with a higher expression of CD44, suggesting that these cells acquired a memory phenotype. Recent reports have suggested that the CD28/CD86 axis in a tumor context is necessary to prevent final CD8 T cell exhaustion and its accumulation (79).. Moreover, this axis rewires the exhaustion trajectory in CD8 T cells, leading to a more central memory phenotype rather than an exhausted phenotype (80). In addition, the tight contact between CD8 T cells and cDC1 in the tumor stroma has been reported as one of the main drivers of tumor rejection in mouse models (73). Our results showed that innate immune training on DCs induced by CTB promoted robust recruitment of these cells at the stimulation site and tumor tissue, with higher expression of CD86, which could directly impact CD8 T cell phenotype. This interaction benefited tumor-challenged mice, emphasizing that this approach could be used as an immunotherapeutic strategy against solid tumors. Furthermore, tumor antigen immunization preceded by innate immune training could be used as combinatorial therapy for current immune checkpoint blockade therapy. Since it reverts functional compromise in CD8 T cells, it could revert immune checkpoint blockade resistance.

Previously described training inductors such as β-glucan or LPS (10, 16, 58) cannot used as adjuvants in vaccine formulations. In contrast, CTB is present in the cholera vaccine and is currently approved for human use (81). The data presented here sustain that CTB, besides its adjuvant role, could be used as an innate training inducer with activity on dendritic cells. The potential use of adjuvants as training stimuli should be further explored since these molecules might not only be involved in prime-boosting adaptive immunity but could also be associated with innate immune training recall and innate immunity protection.

## Data availability statement

The raw data supporting the conclusions of this article will be made available by the authors, without undue reservation.

## Ethics statement

The animal study was approved by The Scientific and Ethics Committee of the Instituto Mexicano del Seguro Social. The study was conducted in accordance with the local legislation and institutional requirements.

## Author contributions

AT-S: Formal Analysis, Investigation, Methodology, Writing – original draft, Writing – review & editing. JG: Formal Analysis, Investigation, Methodology, Writing – original draft, Writing – review & editing. SM-C: Validation, Visualization, Writing – original draft, Writing – review & editing. OC-E: Validation, Visualization, Writing – original draft, Writing – review & editing. LB: Conceptualization, Funding acquisition, Writing – original draft, Writing – review & editing.

## References

[1] Palgen JL Feraoun Y Dzangué-Tchoupou G Joly C Martinon F Le Grand R. Optimize prime/boost vaccine strategies: Trained immunity as a new player in the game. Front Immunol. (2021) 12. doi: 10.3389/fimmu.2021.612747 PMC7982481 33763063

[2] Netea MG Latz E Mills KHG O’Neill LAJ. Innate immune memory: A paradigm shift in understanding host defense. Nat Immunol. (2015) 16:675–9. doi: 10.1038/ni.3178 26086132

[3] O’Sullivan TE Sun JC Lanier LL. Natural killer cell memory. Immunity. (2015) 43:634–45. doi: 10.1016/j.immuni.2015.09.013 PMC4621966 26488815

[4] MacLeod MKL Kappler JW Marrack P. Memory CD4 T cells: Generation, reactivation and re-assignment. Immunology. (2010) 130:10–5. doi: 10.1111/j.1365-2567.2010.03260.x PMC2855788 20331469

[5] Veiga-Fernandes H Walter U Bourgeois C McLean A Rocha B. Response of na.ve and 521 memory CD8+ T cells to antigen stimulation *in vivo* . Nat Immunol. (2000) 1):47–53. doi: 10.1038/76907 10881174

[6] Hu Z Lu SH Lowrie DB Fan XY. Trained immunity: A Yin-Yang balance. MedComm. MedComm Wiley. (2022) 3(1):e121. doi: 10.1002/mco2.121 PMC8906449 35281787

[7] Verma D Parasa VR Raffetseder J Martis M Mehta RB Netea M. Anti-mycobacterial activity correlates with altered DNA methylation pattern in immune cells from BCG-vaccinated subjects. Sci Rep. (2017) 7(1):12305. doi: 10.1038/s41598-017-12110-2 28951586 PMC5615063

[8] Mehta S Jeffrey KL. Beyond receptors and signaling: Epigenetic factors in the regulation of innate immunity. Immunol Cell Biol. (2015) 93:233–44. doi: 10.1038/icb.2014.101 PMC4885213 25559622

[9] Quintin J Saeed S Martens JHA Giamarellos-Bourboulis EJ Ifrim DC Logie C. Candida albicans infection affords protection against reinfection via functional reprogramming of monocytes. Cell Host Microbe. (2012) 12:223–32. doi: 10.1016/j.chom.2012.06.006 PMC3864037 22901542

[10] Saeed S Quintin J Kerstens HHD Rao NA Aghajanirefah A Matarese F. Epigenetic programming of monocyte-to-macrophage differentiation and trained innate immunity. Sci (1979). (2014) 345(6204):1251086. doi: 10.1126/science.1251086 PMC4242194 25258085

[11] Domínguez-Andrées J Dos Santos JC Bekkering S Mulder WJM van der Meer JWM Riksen NP. Trained immunity: Adaptation within innate immune mechanisms. Physiol Rev. (2023) 103:313–46. doi: 10.1152/physrev.00031.2021 35981301

[12] Netea MG Domínguez-Andrés J Barreiro LB Chavakis T Divangahi M Fuchs E. Defining trained immunity and its role in health and disease. Nat Rev Immunol. (2020) 20:375–88. doi: 10.1038/s41577-020-0285-6 PMC7186935 32132681

[13] Kleinnijenhuis J Quintin J Preijers F Joosten LAB Ifrim DC Saeed S. Bacille Calmette-Guérin induces NOD2-dependent nonspecific protection from reinfection via epigenetic reprogramming of monocytes. Proc Natl Acad Sci U S A. (2012) 109:17537–42. doi: 10.1073/pnas.1202870109 PMC3491454 22988082

[14] Bekkering S Dominguez-Andres J Joosten LAB Riksen NP Netea MG. Trained immunity: Reprogramming innate immunity in health and disease. Annu Rev Immunol. (2021) 39:667–93. doi: 10.1146/annurev-immunol-102119-073855 33637018

[15] Riksen NP Netea MG. Immunometabolic control of trained immunity. Mol Aspects Med. (2021) 77(100897). doi: 10.1016/j.mam.2020.100897 PMC7466946 32891423

[16] Cheng SC Quintin J Cramer RA Shepardson KM Saeed S Kumar V. MTOR- and HIF-1α-mediated aerobic glycolysis as metabolic basis for trained immunity. Sci (1979). (2014) 345(6204):1250684. doi: 10.1126/science.1250684 PMC4226238 25258083

[17] Arts RJW Carvalho A La Rocca C Palma C Rodrigues F Silvestre R. Immunometabolic pathways in BCG-induced trained immunity. Cell Rep. (2016) 17:2562–71. doi: 10.1016/j.celrep.2016.11.011 PMC5177620 27926861

[18] Kaufmann E Sanz J Dunn JL Khan N Mendonça LE Pacis A. BCG educates hematopoietic stem cells to generate protective innate immunity against tuberculosis. Cell. (2018) 172:176–90.e19. doi: 10.1016/j.cell.2017.12.031. 29328912

[19] Mitroulis I Ruppova K Wang B Chen LS Grzybek M Grinenko T. Modulation of myelopoiesis progenitors is an integral component of trained immunity. Cell. (2018) 172:147–61.e12. doi: 10.1016/j.cell.2017.11.034 29328910 PMC5766828

[20] Kleinnijenhuis J Van Crevel R Netea MG. Trained immunity: Consequences for the heterologous effects of BCG vaccination. Trans R Soc Trop Med Hyg. (2014) 109:29–35. doi: 10.1093/trstmh/tru168 25573107

[21] Cirovic B de Bree LCJ Groh L Blok BA Chan J van der Velden WJFM. BCG vaccination in humans elicits trained immunity via the hematopoietic progenitor compartment. Cell Host Microbe. (2020) 28:322–334.e5. doi: 10.1016/j.chom.2020.05.014 32544459 PMC7295478

[22] de Laval B Maurizio J Kandalla PK Brisou G Simonnet L Huber C. C/EBPβ-dependent epigenetic memory induces trained immunity in hematopoietic stem cells. Cell Stem Cell. (2020) 26:657–674.e8. doi: 10.1016/j.stem.2020.01.017 32169166

[23] Garcia-Valtanen P Guzman-Genuino RM Williams DL Hayball JD Diener KR. Evaluation of trained immunity by β-1, 3 (d)-glucan on murine monocytes *in vitro* and duration of response *in vivo* . Immunol Cell Biol. (2017) 95:601–10. doi: 10.1038/icb.2017.13 PMC5550561 28228641

[24] Rizzetto L Ifrim DC Moretti S Tocci N Cheng SC Quintin J. Fungal chitin induces trained immunity in human monocytes during cross-talk of the host with Saccharomyces cerevisiae. J Biol Chem. (2016) 291:7961–72. doi: 10.1074/jbc.M115.699645 PMC4825003 26887946

[25] Hole CR Wager CML Castro-Lopez N Campuzano A Cai H Wozniak KL. Induction of memory-like dendritic cell responses *in vivo* . Nat Commun. (2019) 10:1–13. doi: 10.1038/s41467-019-10486-5 31273203 PMC6609631

[26] del Rio ML Rodriguez-Barbosa JI Kremmer E Förster R. CD103– and CD103+ Bronchial lymph node dendritic cells are specialized in presenting and cross-presenting innocuous antigen to CD4+ and CD8+ T cells. J Immunol. (2007) 178:6861–6. doi: 10.4049/jimmunol.178.11.6861 17513734

[27] Roberts EW Broz ML Binnewies M Headley MB Nelson AE Wolf DM. Critical role for CD103+/CD141+ Dendritic cells bearing CCR7 for tumor antigen trafficking and priming of T cell immunity in melanoma. Cancer Cell. (2016) 30:324–36. doi: 10.1016/j.ccell.2016.06.003 PMC5374862 27424807

[28] Dudziak D Kamphorst AO Heidkamp GF Buchholz VR Trumpfheller C Yamazaki S. Differential antigen processing by dendritic cell subsets *in vivo* . Sci (1979). (2007) 315:107–11. doi: 10.1126/science.1136080 17204652

[29] Steinman RM Idoyaga J. Features of the dendritic cell lineage. Immunol Rev. (2010) 234:5–17. doi: 10.1111/j.0105-2896.2009.00888.x 20193008

[30] Menezes S Melandri D Anselmi G Perchet T Loschko J Dubrot J. The Heterogeneity of Ly6C^hi^ Monocytes Controls Their Differentiation into iNOS^+^ Macrophages or Monocyte-Derived Dendritic Cells. Immunity. (2016) 45:1205–18. doi: 10.1016/j.immuni.2016.12.001 PMC5196026 28002729

[31] Zhan Y Xu Y Seah S Brady JL Carrington EM Cheers C. Resident and monocyte-derived dendritic cells become dominant IL-12 producers under different conditions and signaling pathways. J Immunol. (2010) 185:2125–33. doi: 10.4049/jimmunol.0903793 20644172

[32] Sharma MD Rodriguez PC Koehn BH Baban B Cui Y Guo G. Activation of p53 in Immature Myeloid Precursor Cells Controls Differentiation into Ly6c+CD103+ Monocytic Antigen-Presenting Cells in Tumors. Immunity. (2018) 48(1):91–106.e6. doi: 10.1016/j.immuni.2017.12.014 29343444 PMC6005382

[33] Antonio-Herrera L Badillo-Godinez O Medina-Contreras O Tepale-Segura A García-Lozano A Gutierrez-Xicotencatl L. The nontoxic cholera B subunit is a potent adjuvant for intradermal DC-targeted vaccination. Front Immunol. (2018) 9. doi: 10.3389/fimmu.2018.02212 PMC6171476 30319653

[34] León-Letelier RA Castro-Medina DI Badillo-Godinez O Tepale-Segura A Huanosta-Murillo E Aguilar-Flores C. Induction of progenitor exhausted tissue-resident memory CD8+ T cells upon salmonella typhi porins adjuvant immunization correlates with melanoma control and anti-PD-1 immunotherapy cooperation. Front Immunol. (2020) 11. doi: 10.3389/fimmu.2020.583382 PMC7682137 33240271

[35] Dähling S Mansilla AM Knöpper K Grafen A Utzschneider DT Ugur M. Type 1 conventional dendritic cells maintain and guide the differentiation of precursors of exhausted T cells in distinct cellular niches. Immunity. (2022) 55:656–70.e8. doi: 10.1016/j.immuni.2022.03.006 35366396

[36] Merad M Sathe P Helft J Miller J Mortha A. The dendritic cell lineage: ontogeny and function of dendritic cells and their subsets in the steady state and the inflamed setting. Annu Rev Immunol. (2013) 31:563–604. doi: 10.1146/annurev-immunol-020711-074950 23516985 PMC3853342

[37] Cabeza-Cabrerizo M Cardoso A Minutti CM Pereira da Costa M Reis Sousa C. Dendritic cells revisited. Annu Rev Immunol. (2021) 26(39):131–66. doi: 10.1146/annurev-immunol-061020-053707 33481643

[38] Diao J Gu H Tang M Zhao J Cattral MS. Tumor dendritic cells (DCs) derived from precursors of conventional DCs are dispensable for intratumor CTL responses. J Immunol. (2018) 201:1306–14. doi: 10.4049/jimmunol.1701514 PMC6077850 29997124

[39] Bayerl F Meiser P Donakonda S Hirschberger A Lacher SB Pedde AM. Tumor-derived prostaglandin E2 programs cDC1 dysfunction to impair intratumoral orchestration of anti-cancer T cell responses. Immunity. (2023) 56:1341–58.e11. doi: 10.1016/j.immuni.2023.05.011 37315536

[40] Cabeza-Cabrerizo M van Blijswijk J Wienert S Heim D Jenkins RP Chakravarty P. Tissue clonality of dendritic cell subsets and emergency DCpoiesis revealed by multicolor fate mapping of DC progenitors. Sci Immunol. (2019) 4:1–14. doi: 10.1126/sciimmunol.aaw1941 PMC6420147 30824528

[41] Cheong JG Ravishankar A Sharma S Parkhurst CN Grassmann SA Wingert CK. Epigenetic memory of coronavirus infection in innate immune cells and their progenitors. Cell. (2023) 186(18):3882–902.e24. doi: 10.1016/j.cell.2023.07.019 37597510 PMC10638861

[42] Jeljeli M Riccio LGC Chouzenoux S Moresi F Toullec L Doridot L. Macrophage immune memory controls endometriosis in mice and humans. Cell Rep. (2020) 33(5):108325. doi: 10.1016/j.celrep.2020.108325 33147452

[43] Kalafati L Kourtzelis I Schulte-Schrepping J Li X Hatzioannou A Grinenko T. Innate immune training of granulopoiesis promotes anti-tumor activity. Cell. (2020) 183:771–85.e12. doi: 10.1016/j.cell.2020.09.058 33125892 PMC7599076

[44] Arts RJW Moorlag SJCFM Novakovic B Li Y Wang SY Oosting M. BCG Vaccination Protects against Experimental Viral Infection in Humans through the Induction of Cytokines Associated with Trained Immunity. Cell Host Microbe. (2018) 23:89–100.e5. doi: 10.1016/j.chom.2017.12.010 29324233

[45] Tarancón R Domínguez-Andrés J Uranga S Ferreira AV Groh LA Domenech M. New live attenuated tuberculosis vaccine MTBVAC induces trained immunity and confers protection against experimental lethal pneumonia. PLoS Pathog. (2020) 16(4):e1008404. doi: 10.1371/journal.ppat.1008404 32240273 PMC7117655

[46] Rivas MN Ebinger JE Wu M Sun N Braun J Sobhani K. BCG vaccination history associates with decreased SARS-CoV-2 seroprevalence across a diverse cohort of health care workers. J Clin Invest. (2021) 131(2):e145157. doi: 10.1172/JCI145157 33211672 PMC7810479

[47] Meza-Sánchez D Pérez-Montesinos G Sánchez-García J Moreno J Bonifaz LC. Intradermal immunization in the ear with cholera toxin and its non-toxic ?? subunit promotes efficient Th1 and Th17 differentiation dependent on migrating DCs. Eur J Immunol. (2011) 41:2894–904. doi: 10.1002/eji.201040997 21792876

[48] León B Ardavín C. Monocyte migration to inflamed skin and lymph nodes is differentially controlled by L-selectin and PSGL-1. Blood. (2008) 111:3126–30. doi: 10.1182/blood-2007-07-100610 18184867

[49] León B López-Bravo M Ardavín C. Monocyte-Derived Dendritic Cells Formed at the Infection Site Control the Induction of Protective T Helper 1 Responses against Leishmania. Immunity. (2007) 26:519–31. doi: 10.1016/j.immuni.2007.01.017 17412618

[50] Parikh SV Malvar A Shapiro J Turman JM Song H Alberton V. A novel inflammatory dendritic cell that is abundant and contiguous to T cells in the kidneys of patients with lupus nephritis. Front Immunol. (2021) 12. doi: 10.3389/fimmu.2021.621039 PMC7919935 33659005

[51] Saxena M Bhardwaj N. Turbocharging vaccines: emerging adjuvants for dendritic cell based therapeutic cancer vaccines. Curr Opin Immunol. (2017) 47:35–43. doi: 10.1016/j.coi.2017.06.003 28732279 PMC5626599

[52] Holmgren J Lycke N Czerkinsky C. Cholera toxin and cholera B subunit as oral—mucosal adjuvant and antigen vector systems. Vaccine. (1993) 11:1179–84. doi: 10.1016/0264-410X(93)90039-Z 8256498

[53] Baldauf KJ Royal JM Hamorsky KT Matoba N. Cholera toxin B: One subunit with many pharmaceutical applications. Toxins. MDPI AG. (2015) 7:974–96. doi: 10.3390/toxins7030974 PMC4379537 25802972

[54] Stothers CL Burelbach KR Owen AM Patil NK McBride MA Bohannon JK. β-glucan induces distinct and protective innate immune memory in differentiated macrophages. J Immunol. (2021) 207:2785–98. doi: 10.4049/jimmunol.2100107 PMC8612974 34740960

[55] Low R Ha SD Sleapnicov N Maneesh P Kim SO. Prolonged inhibition of the MEK1/2-ERK signaling axis primes interleukin-1 beta expression through histone 3 lysine 9 demethylation in murine macrophages. Int J Mol Sci. (2023) 24(19):14428.S. doi: 10.20944/preprints202308.1764.v1 37833877 PMC10572145

[56] Bekkering S Blok BA Joosten LAB Riksen NP Van Crevel R Netea MG. *In Vitro* experimental model of trained innate immunity in human primary monocytes. Clin Vaccine Immunol. (2016) 23:926–33. doi: 10.1128/CVI.00349-16 PMC5139603 27733422

[57] Hogquist KA Jameson SC Heath WR Howard JL Bevan MJ. Carbone FR. T cell receptor antagonist peptides induce positive selection. Cell. (1994) 76(1):17–27. doi: 10.1016/0092-8674(94)90169-4 8287475

[58] Priem B van Leent MMT Teunissen AJP Sofias AM Mourits VP Willemsen L. Trained immunity-promoting nanobiologic therapy suppresses tumor growth and potentiates checkpoint inhibition. Cell. (2020) 183:786–801.e19 doi: 10.1016/j.cell.2020.09.059. 33125893 PMC8074872

[59] Phongsisay V Hara H Yoshida H. Evidence for TLR4 and FcRg – CARD9 activation by cholera toxin B subunit and its direct bindings to TREM2 and LMIR5 receptors. Mol Immunol. (2015) 66(2):463–71. doi: 10.1016/j.molimm.2015.05.008 26021803

[60] Bono C Guerrero P Jordán-Pla A Erades A Salomonis N Grimes HL. GM-CSF programs hematopoietic stem and progenitor cells during candida albicans vaccination for protection against reinfection. Front Immunol. (2021) 12. doi: 10.3389/fimmu.2021.790309 PMC8715000 34975887

[61] Borriello F Iannone R Di Somma S Loffredo S Scamardella E Galdiero MR. GM-CSF and IL-3 modulate human monocyte TNF-α production and renewal in *in vitro* models of trained immunity. Front Immunol. (2017) 7. doi: 10.3389/fimmu.2016.00680 PMC5237654 28138327

[62] Chen L Flies DB. Molecular mechanisms of T cell co-stimulation and co-inhibition. Nat Rev Immunol. (2013) 13:227–42. doi: 10.1038/nri3405 PMC3786574 23470321

[63] Thomas IJ Petrich de Marquesini LG Ravanan R Smith RM Guerder S Flavell RA. CD86 has sustained costimulatory effects on CD8 T cells. J Immunol. (2007) 179(9):5936–46. doi: 10.4049/jimmunol.179.9.5936 PMC2629533 17947667

[64] Compans ER Cooper G Honjo AT Koprowski H Melchers PF Oldstone M. From Innate Immunity to Immunological Memory. Current Topics Microbiol Immunol. (2006) 311:17–58.

[65] Duraiswamy J Turrini R Minasyan A Barras D Crespo I Grimm AJ. Myeloid antigen-presenting cell niches sustain antitumor T cells and license PD-1 blockade via CD28 costimulation. Cancer Cell. (2021) 39:1623–42.e20. doi: 10.1016/j.ccell.2021.10.008 34739845 PMC8861565

[66] Fan M Chen S Weng Y Li X Jiang Y Wang X. Ciprofloxacin promotes polarization of CD86+CD206- macrophages to suppress liver cancer. Oncol Rep. (2020) 44:91–102. doi: 10.3892/or 32377744 PMC7251753

[67] Li F Tian Z. The liver works as a school to educate regulatory immune cells. Cell Mol Immunol. (2013) 10:292–302. doi: 10.1038/cmi.2013.7 23604044 PMC4003213

[68] Bousso P Robey E. Dynamics of CD8 + T cell priming by dendritic cells in intact lymph nodes. Nat Immunol. (2003) 4(6):579–85. doi: 10.1038/ni928 12730692

[69] Miller MJ Wei SH Parker I Cahalan MD. Two-photon imaging of lymphocyte motility and antigen response in intact lymph node. Science (1979). (2002) 296:1869–73. doi: 10.1126/science.1070051 12016203

[70] Steinman RM. The Dendritic Cell System and its role in immunogenicity. Annu Rev Immunol. (1991) 9:271–96. doi: 10.1146/annurev.iy.09.040191.001415 1910679

[71] Sangsuwan R Thuamsang B Pacifici N Allen R Han H Miakicheva S. Lactate exposure promotes immunosuppressive phenotypes in innate immune cells. Cell Mol Bioeng. (2020) 13:541–57. doi: 10.1007/s12195-020-00652-x PMC7596145 33184582

[72] Wang JX Choi SYC Niu X Kang N Xue H Killam J. Lactic acid and an acidic tumor microenvironment suppress anticancer immunity. Int J Mol Sci. (2020) 21:1–14. doi: 10.3390/ijms21218363 PMC7664620 33171818

[73] Meiser P Knolle MA Hirschberger A de Almeida GP Bayerl F Lacher S. A distinct stimulatory cDC1 subpopulation amplifies CD8+ T cell responses in tumors for protective anti-cancer immunity. Cancer Cell. (2023) 41:1498–1515.e10. doi: 10.1016/j.ccell.2023.06.008 37451271

[74] Wylie B Read J Buzzai AC Wagner T Troy N Syn G. CD8+XCR1neg dendritic cells express high levels of toll-like receptor 5 and a unique complement of endocytic receptors. Front Immunol. (2019) 10. doi: 10.3389/fimmu.2018.02990 PMC6343586 30700986

[75] Miller JC Brown BD Shay T Gautier EL Jojic V Cohain A. Deciphering the transcriptional network of the dendritic cell lineage. Nat Immunol. (2012) 13:888–99. doi: 10.1038/ni.2370 PMC3985403 22797772

[76] Schenkel JM Herbst RH Canner D Li A Hillman M Shanahan SL. Conventional type I dendritic cells maintain a reservoir of proliferative tumor-antigen specific TCF-1+ CD8+ T cells in tumor-draining lymph nodes. Immunity. (2021) 54:2338–2353.e6. doi: 10.1016/j.immuni.2021.08.026 34534439 PMC8604155

[77] Wesa A Kalinski P Zyj W Kirkwood JM Tatsumi T Storkus WJ. Polarized type-1 dendritic cells (DC1) producing high levels of IL-12 family members rescue patient T H 1-type antimelanoma CD4 + T cell responses *in vitro* . J Immunotherapy (2006) 30(1):75–82. doi: 10.1097/01.cji.0000211316.15278.6e 17198085

[78] Mineharu Y Muhammad AG Yagiz K Candolfi M Kroeger KM Xiong W. Gene therapy-mediated reprogramming tumor infiltrating T cells using IL-2 and inhibiting NF-κB signaling improves the efficacy of immunotherapy in a brain cancer model. Neurotherapeutics. (2012) 9:827–43. doi: 10.1007/s13311-012-0144-7 PMC3480576 22996231

[79] Humblin E Korpas I Lu J Filipescu D van der Heide V Goldstein S. Sustained CD28 costimulation is required for self-renewal and differentiation of TCF-1 + PD-1 + CD8 T cells. Sci Immunol. (2023) 8(86). doi: 10.1126/sciimmunol.adg0878 PMC10805182 37624910

[80] Hashimoto M Araki K Cardenas MA Li P Jadhav RR Kissick HT. PD-1 combination therapy with IL-2 modifies CD8+ T cell exhaustion program. Nature. (2022) 610:173–81. doi: 10.1038/s41586-022-05257-0 PMC9793890 36171288

[81] Yuki Y Nojima M Kashima K Sugiura K Maruyama S Kurokawa S. Oral MucoRice-CTB vaccine is safe and immunogenic in healthy US adults. Vaccine. (2022) 40:3372–9. doi: 10.1016/j.vaccine.2022.04.051 35484039

