# Supplementary material for: The cholera toxin B subunit induces trained immunity in dendritic cells and promotes CD8 T cell antitumor immunity
Source: Front Immunol. 2024 May 15;15:1362289. doi: 10.3389/fimmu.2024.1362289 (PMC11133619; doi:10.3389/fimmu.2024.1362289)
Supplement: Supplementary file 1 [file DataSheet_1.pdf]

Supplementary Material

1. Supplementary Figures

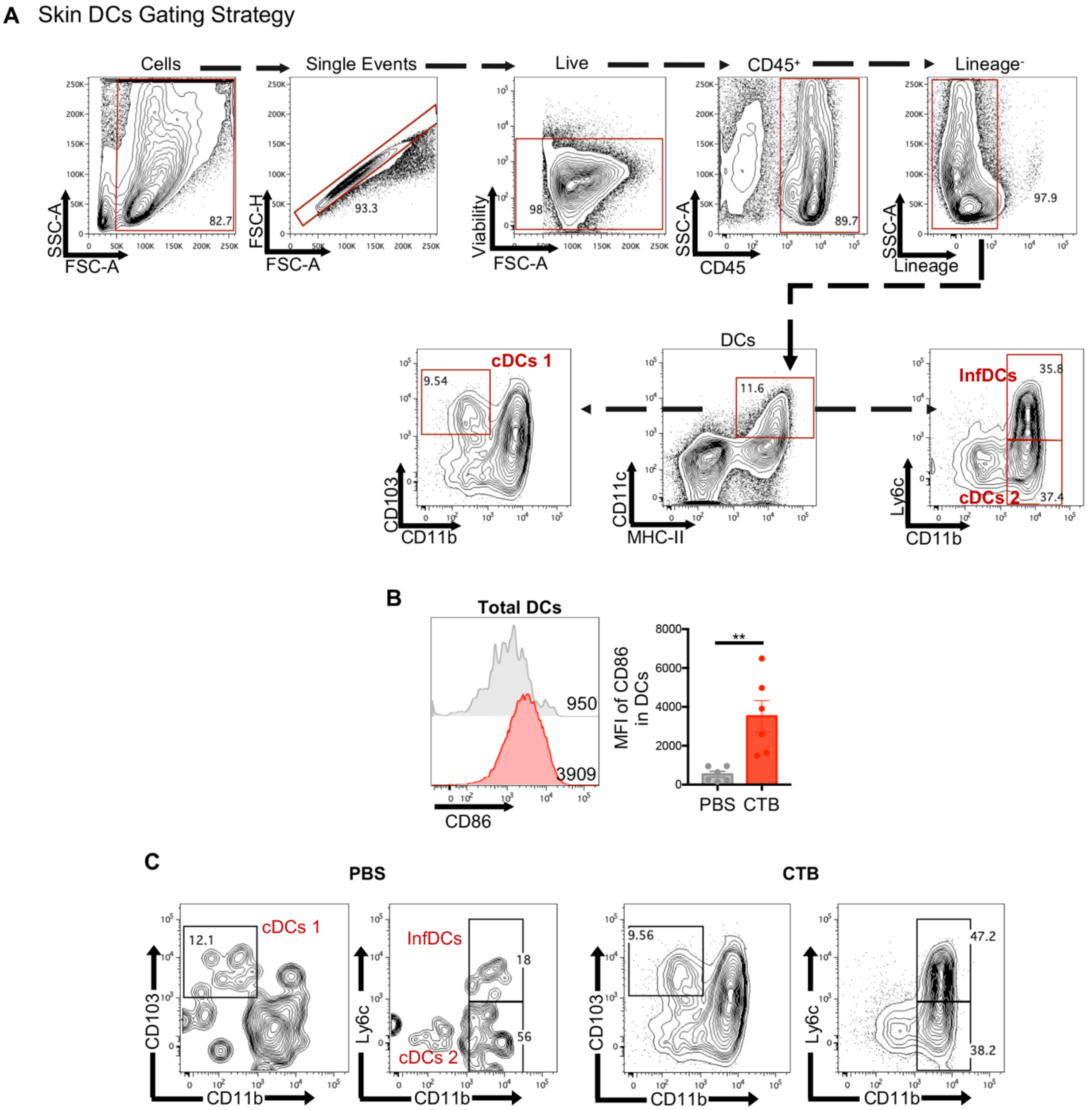

Supplementary Figure 1. Gating strategy used to identify skin DCs subsets. C57BL6 mice were i.d. inoculated with 10 µg of CTB in the ears (5 µg for each ear) or PBS (vehicle). After 7 days, the mice were sacrificed, and the cells from the ear skin were purified. **A** Gating strategy used to identify

DCs. Cells were purified and stained with antibodies to cell surface markers. Small particles (debris) were removed using complexity (SSC-A) and size (FSC-A) parameters, and single events were considered based on height (FSC-H) and area (FSC-A) parameters, followed by Fix Dead Violet viability marker. Subsequently, the CD45^{+} and lineage^{-} (Lin^{-}) populations were selected, from which the CD11c^{+} MHC-II^{+} cells were selected, from this population, three subsets of DCs were identified: cDC1s (CD11b^{-} CD103^{+}); cDC2s (CD11b^{+} Ly6c^{-}) and InfDCs (CD11b^{+} Ly6c^{+}). Lineage markers: CD3: T lymphocytes, CD19: B lymphocytes, TERR-119: erythrocytes/proerythroblasts, and CD49b: NK cells. **B** Representative histograms (left panel) and bar graphs (right panel) of CD86 expression on total DCs. **C** Representative contour plots of the phenotyped DCs subsets for each treatment. For CD86 expression Mean ±SEM, n= 6, data pooled from 2 independent experiments. Kruskal Wallis with Dunn´s Comparison test $**p < 0.005$.

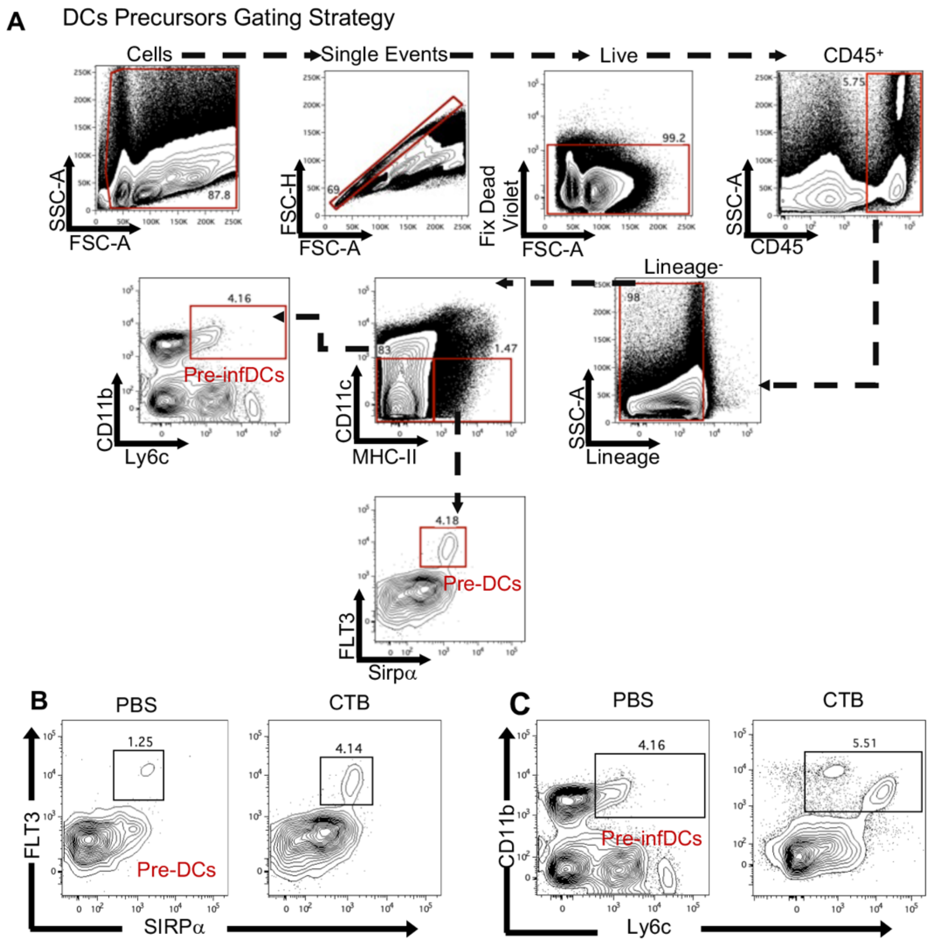

Supplementary Figure 2. Gating strategy followed to identify Pre-DCs and Pre-InfDCs. C57BL6 mice were inoculated i.d. with 10 μg of CTB in the ears (5 μg for each ear) or PBS (vehicle). After 7 days, the ear skin was obtained and the cells were purified. **A** Gating strategy used to identify Pre-DCs; cells were purified and stained with antibodies to cell surface markers. Small particles (debris) were removed using complexity (SSC-A) and size (FSC-A) parameters, and single events were considered based on height (FSC-H) and area (FSC-A) parameters followed by Fix Dead Violet viability marker. Subsequently, the CD45^{+} and Lin^{-} populations were selected. Pre-DCs (FLT3^{+} Sirpα^{+}) were selected from CD11c^{+} MHC-II^{-} subset, and finally Pre-InfDCs (CD11b^{+} Ly6c^{+}) were gated on CD11c^{-} MHC-

II$^-$. Lineage markers: CD3: T lymphocytes, CD19: B lymphocytes, TERR-119: erythrocytes/proerythroblasts, and CD49b: NK cells. **B-C** Representative contour plots of **B** Pre-DCs (FLT3$^+$, SIRPα$^+$) and **C** Pre-InfDCs (CD11b$^+$, Ly6c$^+$).

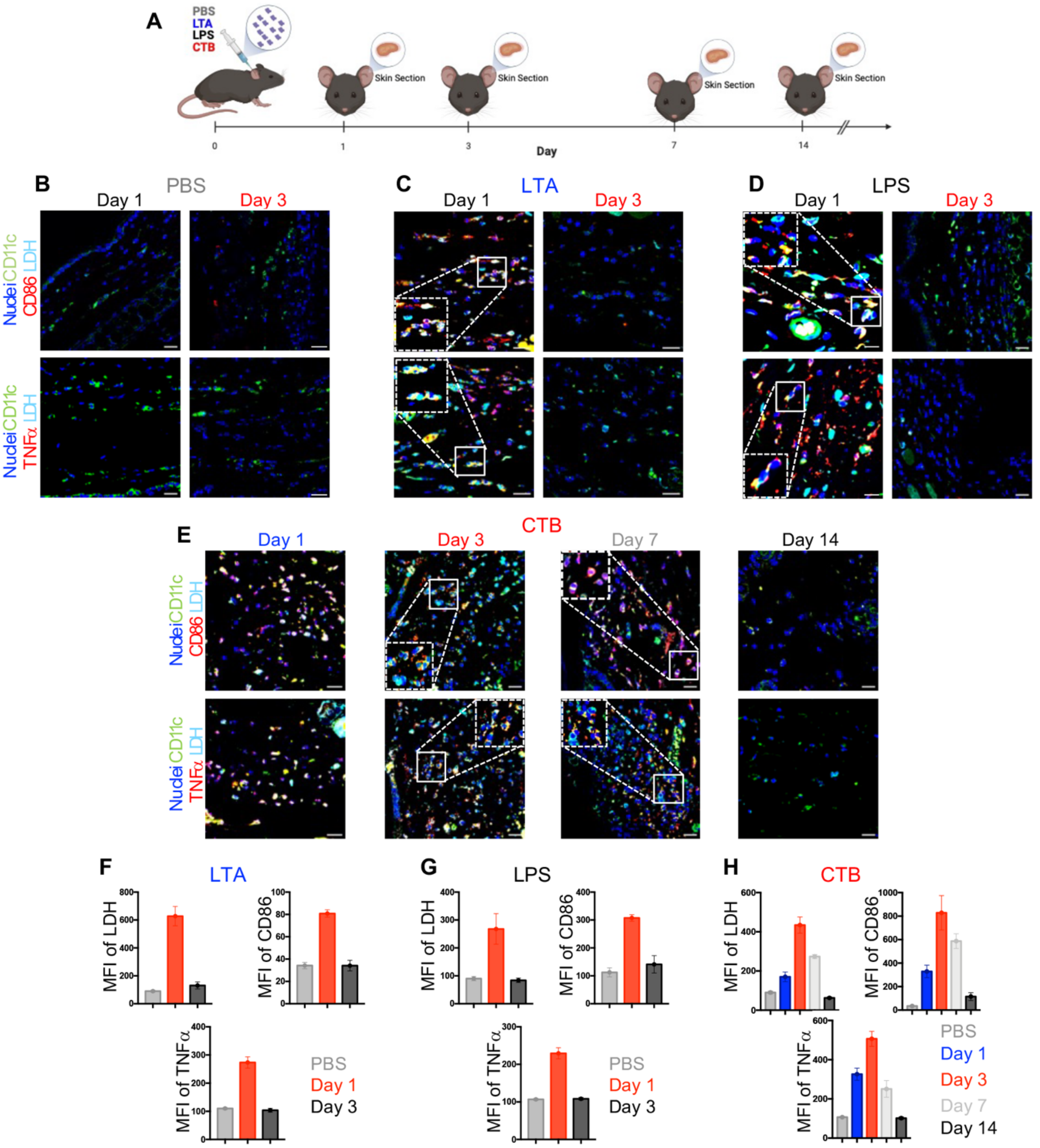

Supplementary Figure 3. CTB immunization promotes and sustains DCs activation and TNFα production. A Schematic representation of the experimental design C57BL6 mice were inoculated i.d. with 10 µg of CTB,10 µg of LPS, 200 µg of purified LTA, or PBS (vehicle). After the time specified, ear skin was collected to obtain histological sections that were stained and analyzed by confocal microscopy. Representative micrographs for LDH, CD86, and TNFα expression after **B** PBS, **C** LTA, **D** LPS, and **E** CTB inoculation at the indicated time (Scale bar 20 µm). Bar graphs of the Mean Fluorescence Intensity (MFI) for LDH, CD86, and TNFα expression in response to **F** LTA, **G** LPS, and **H** CTB administration. White dashed boxes indicate a representative zoomed area from white

boxes. Bar graphs represent an average of three different areas from one mouse at each time. Mouse model figure created with Biorender.

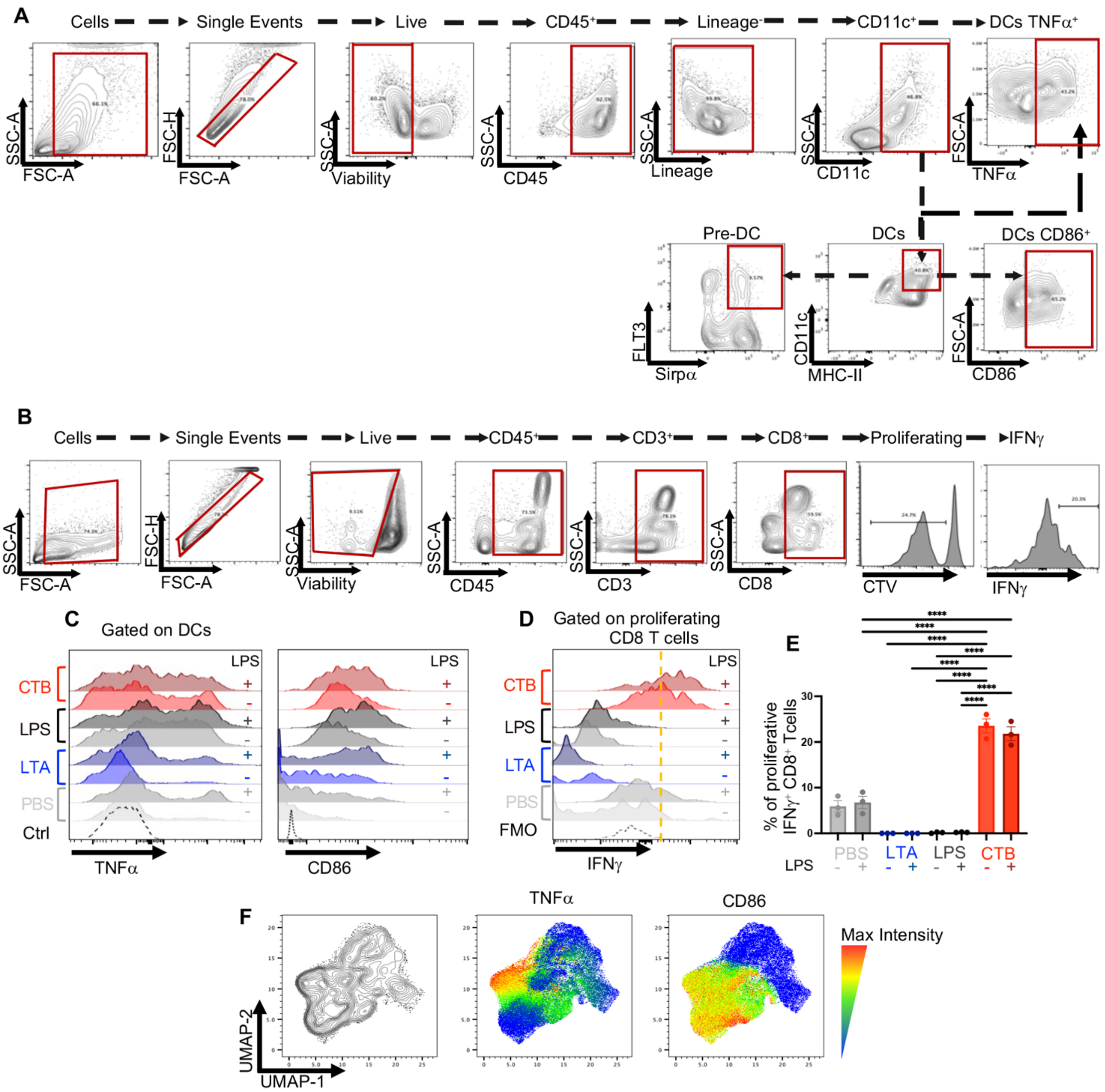

Supplementary Figure 4. CTB administration enhances BMDCs activation and CD8 T cell proliferation. BMDCs were harvested as previously described in figure 3. **A** Gating strategy to identify BMDCs; cells were purified and stained with antibodies to cell surface markers. Small particles (debris) were removed using complexity (SSC-A) and size (FSC-A) parameters, and single events were considered based on height (FSC-H) and area (FSC-A) parameters, followed by Live or dye 568-583 viability marker. Subsequently, the CD45^{+} population and Lin^{-} were selected, from which the CD11c^{+} cells were selected. After CD11c^{+}, MHC-II^{+} subset was considered and finally, TNFα^{+} and CD86^{+} DCs were selected. Alternatively, a Pre-DCs subset was selected from CD11c^{+}, MHC-II^{+} gate (FLT3^{+},

Sirpα^+). **B** To identify $CD8^+$ T cells, we followed the same gate strategy used for the $CD45^+$ subset. Next, the $CD3^+$ population was selected followed by the selection of $CD8^+$ cells. Lastly, to assess T cell proliferation, the Cell Trace Violet (CTV) subset was selected and IFNγ production was determined. **C** Representative histograms of TNFα and CD86 expression in BMDCs for each treatment. **D** Representative histograms for IFNγ expression in proliferative $CD8^+$ T cells in response to different stimuli. **C and D** Autofluorescence control for CD86 or Fluorescence Minus One (FMO) control for INFγ and TNFα are shown in dotted lines. The yellow dotted line shows a reference point for IFNγ expression with respect to the FMO control. **E** Bar graph of the percentage of proliferative IFNγ^+ $CD8^+$ T cells for each condition. Mean ±SEM, n=3. **F** UMAP plots showing dimensionality reduction and clustering of TNFα and CD86 expression for all concatenated samples from DCs subset. The color bar indicates the MFI of TNFα and CD86 expression. Lineage markers: CD3: T lymphocytes, CD19: B lymphocytes, TERR-119: erythrocytes/proerythroblasts, and CD49b: NK cells. Data from pooled bone marrow cells of three mice for each stimulus co-cultured with OT-I cells, in triplicate for each condition. Statistical analysis: one-way ANOVA with Tukey's comparison test ****p<0.0001.

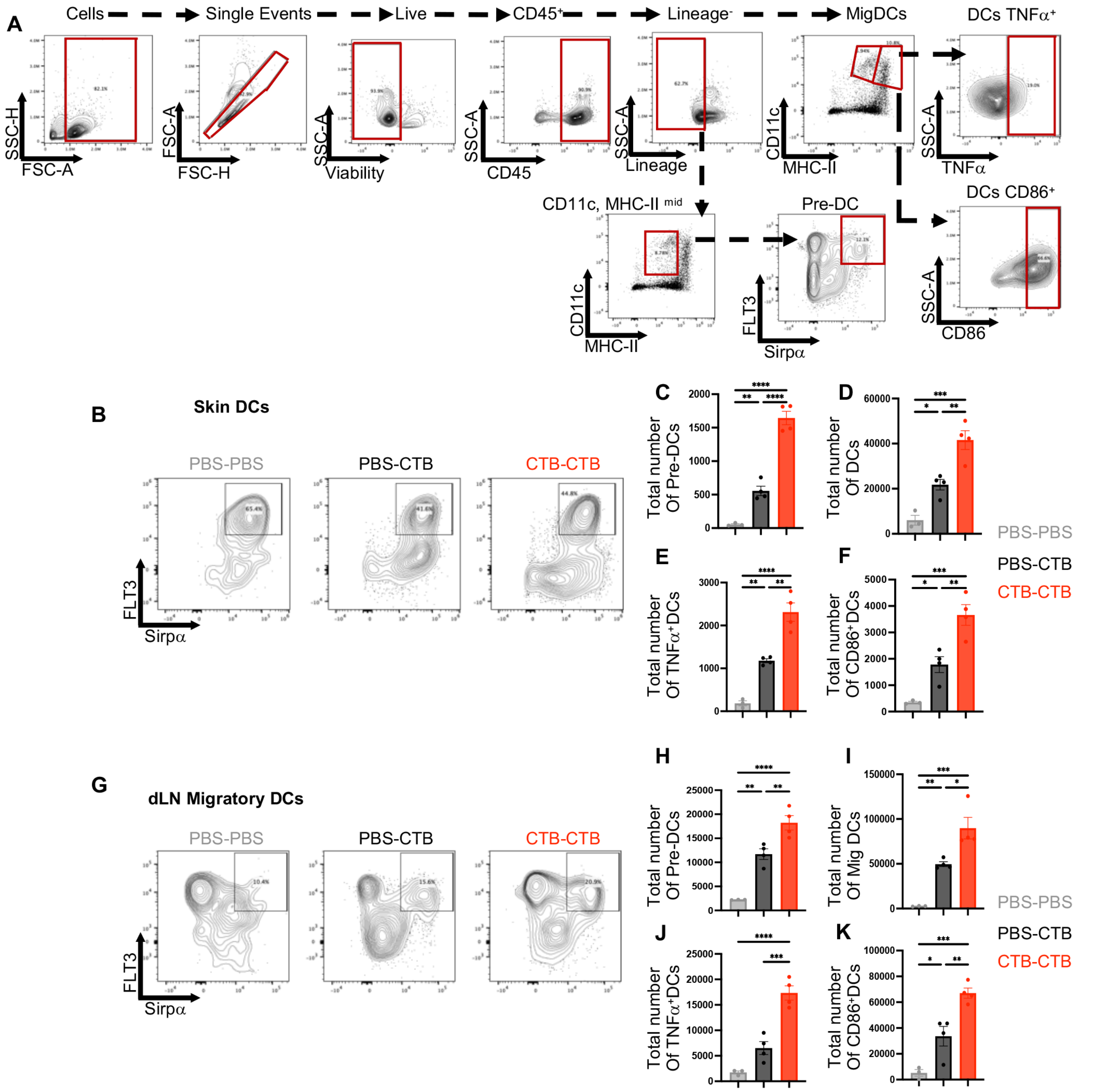

Supplementary Figure 5. Double CTB administration increases PreDCs and DCs with activated phenotype in skin and lymph nodes. C57BL6 mice were inoculated i.d. with 10 µg of CTB (5 µg for each ear) or PBS. After 14 days, the mice were restimulated with the same amount of CTB or PBS, and after 7 days the tissue was obtained and the cells were purified. **A** Gating strategy used to identify PreDCs and DCs in the dLN; cells were purified and stained with antibodies to cell surface markers. Small particles (debris) were removed using complexity (SSC-H) and size (FSC-A) parameters, and single events were considered based on height (FSC-H) and area (FSC-A) parameters, followed by Live or dye 568-583 viability marker. Subsequently, the CD45^{+} and lineage^{-} populations were selected. Pre-DCs (FLT3^{+} Sirpα^{+}) were selected from CD11c^{+} MHC-II^{Mid} subset. To identify Migratory (Mig) DCs, the CD11c^{+} MHC-II^{High} subset was selected, followed by gating of TNFα^{+} and CD86^{+} DCs. **B** Representative Contour plots of pre-DCs in the skin for each condition. Bar graphs of the total number

of **C** Pre-DCs, **D** skin DCs, **E** TNFα$^+$ DCs, **F** CD86$^+$ DCs, for each treatment. **G** Representative contour plots of Pre-DCs in the dLN for each condition. Bar graphs of the total number of **H** Pre-DCs, **I** Migratory DCs, **J** TNFα$^+$ DCs, **K** CD86$^+$ DCs in dLN for each treatment. Total numbers: Mean ±SEM, n= 3, data from one experiment done in triplicate, with 3 mice per group. Statistical Analysis: One-way ANOVA with Tukey's comparison test * P <0.05 **P < 0.005, ***P = 0.0001 ****P<0.0001. Lineage markers: CD3: T lymphocytes, CD19: B lymphocytes, TERR-119: erythrocytes/proerythroblasts, and CD49b: NK cells.

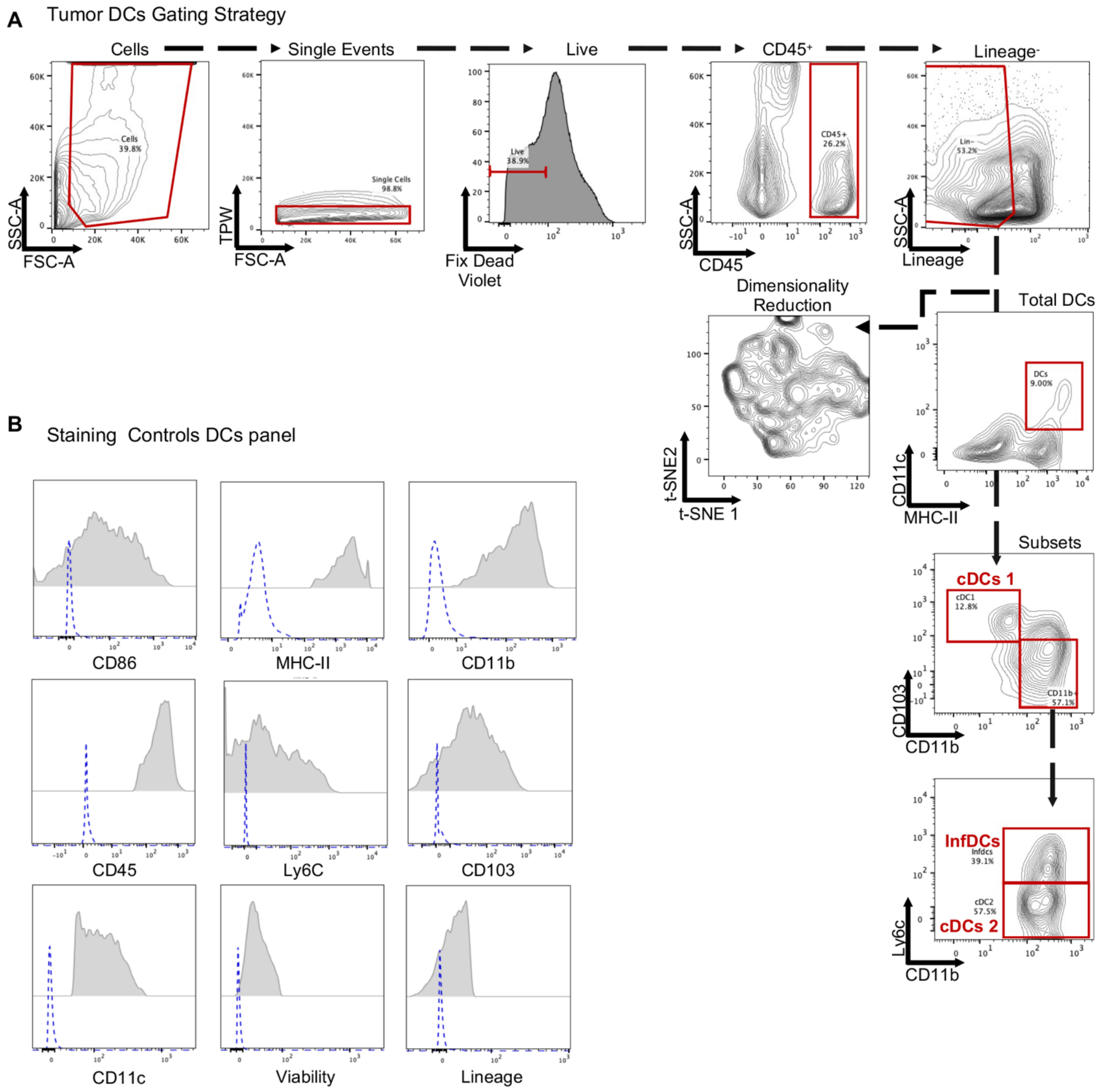

Supplementary Figure 6. Gating strategy to identify tumor-infiltrating DCs. Tumor-infiltrating cells were harvested from tumors obtained as previously described in the mouse model in Figure 4A. **A** Gating strategy used to identify tumor DCs. Cells were stained with antibodies to cell surface markers. Small particles (debris) were removed using complexity (SSC-A) and size (FSC-A)

parameters, single events were considered based on width (Trigger Pulse Width) and area (FSC-A) parameters followed by Fix Dead Violet viability marker. Subsequently, the CD45^{+} and lineage^{-} populations were selected, from which the CD11c^{+} MHC-II^{+} cells were selected, and finally, from this last population, three subsets of DCs cDC1s (CD11b^{-}CD103^{+}); cDC2s (CD11b^{+}, Ly6c^{-}), and InfDCs (CD11b^{+}, Ly6c^{+}), were identified. Lineage markers: CD3: T lymphocytes, CD19: B lymphocytes, TERR-119: Erythrocytes/proerythroblasts, and CD49b: NK cells. **B** Staining controls of tumor DCs panel: stained cells are shown in grey, autofluorescence control for membrane markers are shown in blue dotted lines.

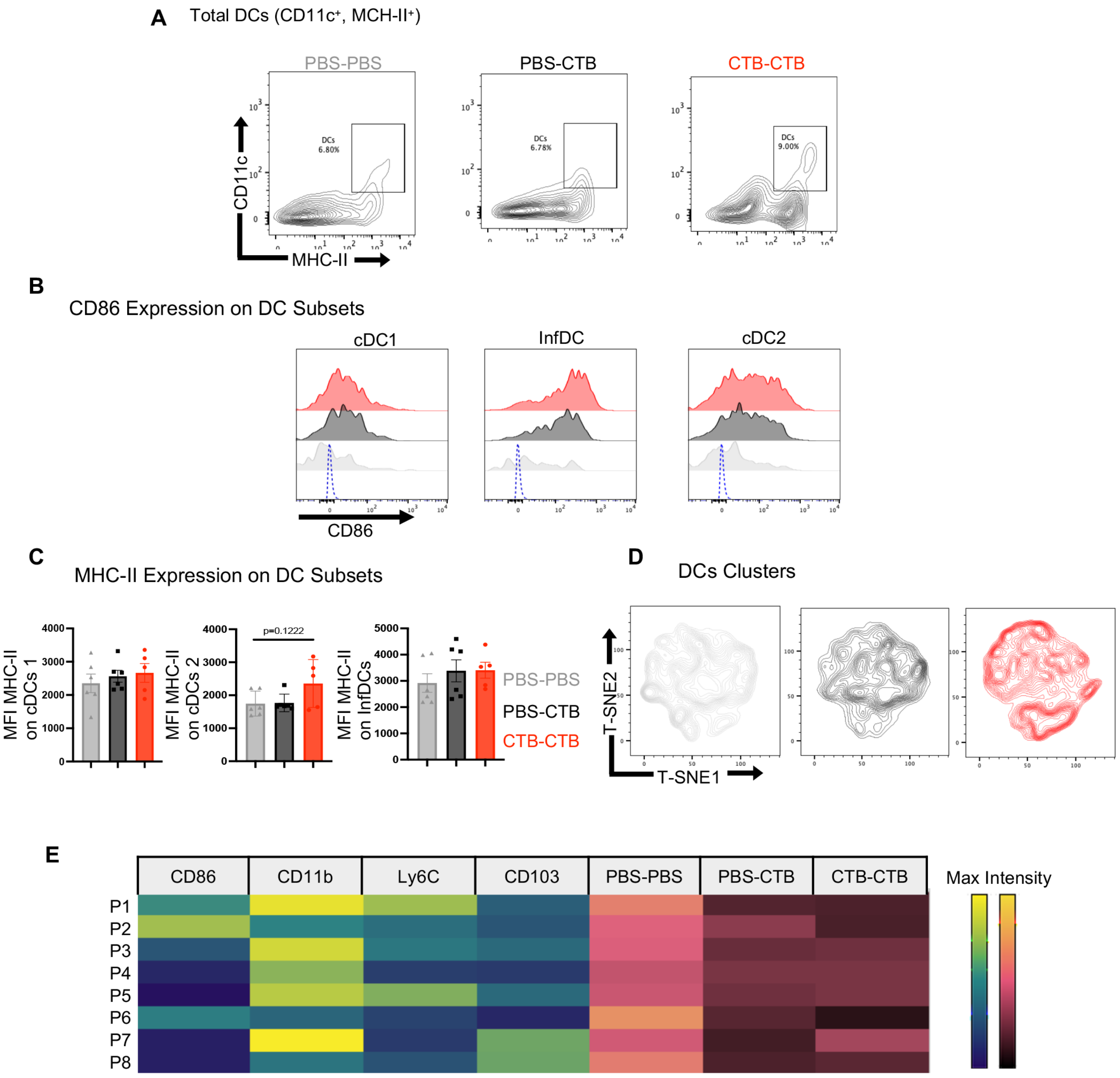

Supplementary Figure 7. MHC-II expression on tumor-infiltrating DCs subsets is not affected by CTB-trained induction in melanoma tumors. Tumor-infiltrating cells were harvested from tumors obtained as previously described in the mouse model in Figure 4A. Subsets and phenotypes of infiltrating DCs were evaluated by multiparametric flow cytometry. **A** Representative Contour plots of an infiltrating of total DCs (CD11c^{+}, MCH-II^{+}), **B** Representative Histograms of CD86 expression on DCs subsets. Blue dotted lines represent autofluorescence control for CD86 **C** Bar graphs showing Mean Fluorescence Intensity (MFI) of MHC-II on DCs subsets **D** t-SNE plots showing dimensionality reduction and clustering among concatenated groups on total DCs. **E** Heatmap showing the MFI (Viridis Scale) of CD86, CD11b, Ly6c and CD103 on FlowSom, differentially enriched clusters from t-SNE dimensionality reduction (P1-P8) and its abundance among treatments (Magma Scale). n= 6 or 5 per group, data pooled from 2 independent experiments, Mean ±SEM. One-way ANOVA with Tukey´s multiple comparison test.

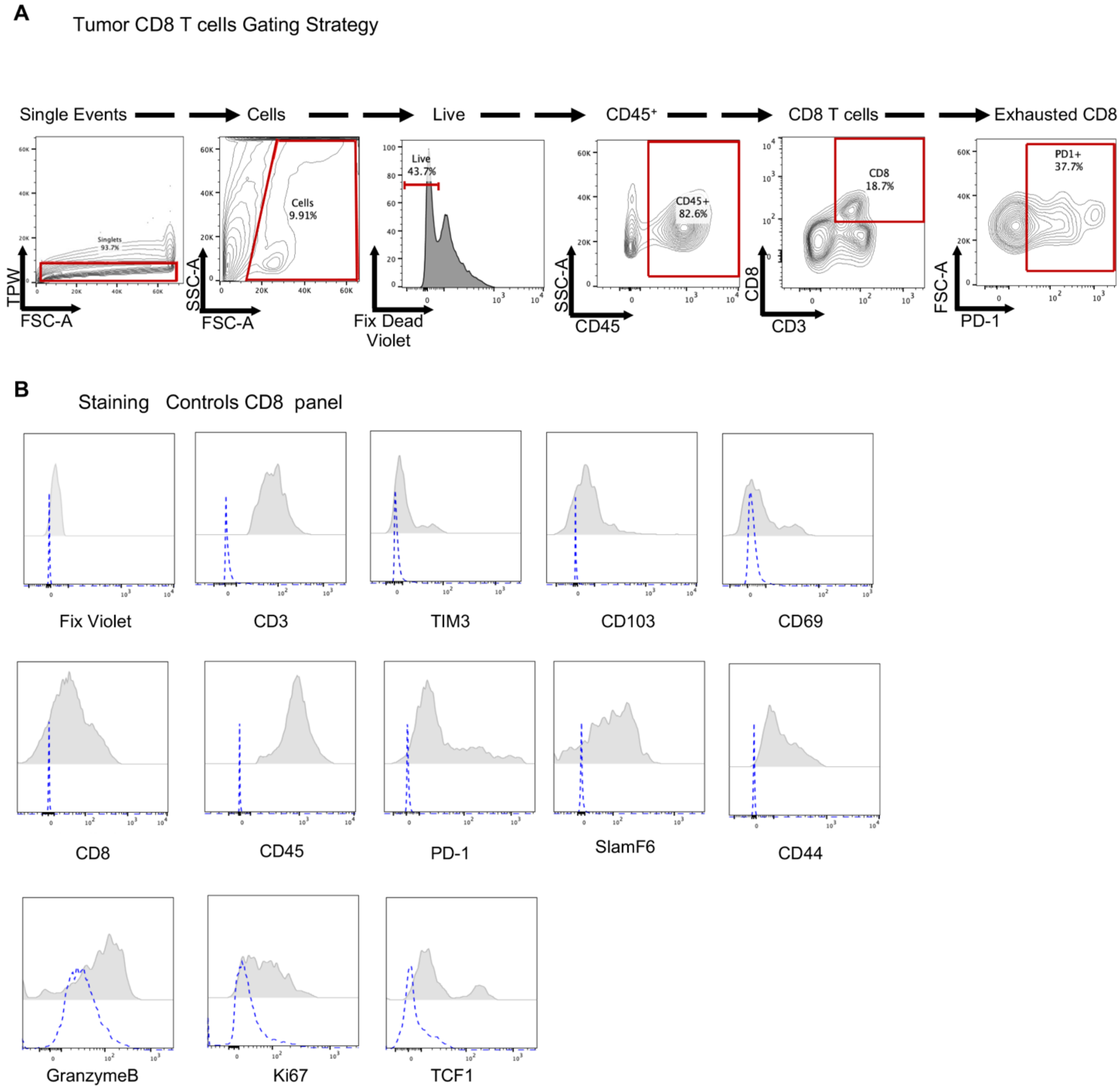

Supplementary Figure 8. Gating strategy followed to identify CD8$^+$ T Lymphocytes. Tumor-infiltrating cells were harvested from tumors obtained as previously described in the mouse model in Figure 4A. **A** Gating strategy to identify CD8$^+$ T lymphocytes. Cells were stained with antibodies to cell surface markers. Single events were considered based on width (Trigger Pulse Width) and area (FSC-A) parameters and small particles (debris) were removed using complexity (SSC-A) and size (FSC-A) parameters, followed by Fix Dead Violet viability marker. Subsequently, the CD45$^+$ population was selected, from which the CD8$^+$ CD3$^+$ population was selected. Finally, exhausted CD8$^+$ lymphocytes were chosen by PD-1 expression. **B** Staining controls of CD8$^+$ T cell panel: stained cells are shown in grey, autofluorescence control for membrane markers, or Fluorescence Minus One (FMO) control for intracellular markers, are shown in blue dotted lines.
